# Green Approach for Rare Earth Element (REE) Recovery from Coal Fly Ash

**DOI:** 10.1021/acs.est.2c09273

**Published:** 2023-03-21

**Authors:** Pan Liu, Simin Zhao, Nan Xie, Lufeng Yang, Qian Wang, Yinghao Wen, Hailong Chen, Yuanzhi Tang

**Affiliations:** † School of Earth and Atmospheric Sciences, Georgia Institute of Technology, 311 Ferst Dr, Atlanta, Georgia 30332, United States; ‡ Woodruff School of Mechanical Engineering, Georgia Institute of Technology, 771 Ferst Dr, Atlanta, Georgia 30332, United States

**Keywords:** rare earth elements, coal fly ash, zeolite, resource recovery, waste management

## Abstract

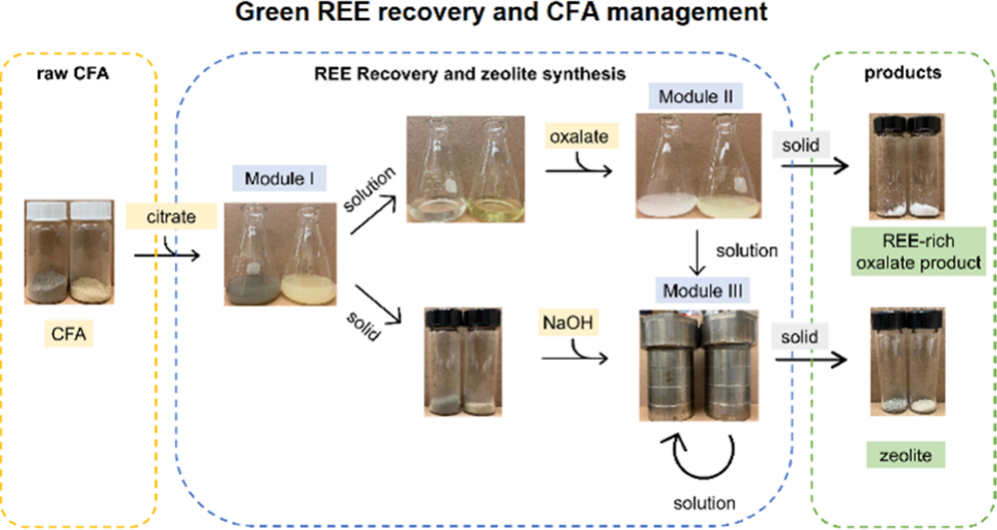

Due to the growing demands of rare earth elements (REEs) and the vulnerability of REEs to potential supply disruption, there have been increasing interests in recovering REEs from waste streams such as coal fly ash (CFA). Meanwhile, CFA as a large industrial waste stream in the United States (U.S.) poses significant environmental and economic burdens. Recovery of REEs from CFA is a promising solution to the REE scarcity issue and also brings opportunities for CFA management. This study demonstrates a green system for REE recovery from Class F and C CFA that consists of three modules: REE leaching using citrate, REE separation and concentration using oxalate, and zeolite synthesis using secondary wastes from Modules I and II. In Module I, ∼10 and 60% REEs were leached from the Class F and C CFA samples, respectively, using citrate at pH 4. In Module II, the addition of oxalate selectively precipitated and concentrated REEs from the leachate via the formation of weddellite (CaC_2_O_4_·2H_2_O), while other trace metals remained in solution. In Module III, zeolite was synthesized using wastes from Modules I and II. This study is characterized by the successful recovery of REEs and upcycling of secondary wastes, which addresses both REE recovery and CFA management challenges.

## Abstract 2

This study develops a green approach for the recovery of rare earth elements from coal fly ash, with the generation of multiple products and upcycling of waste residues.

## 1 Introduction

Rare earth elements (REEs, including the lanthanide elements and yttrium) are widely used in a range of high-tech applications.^1^ Due to the growing demands of REEs and the vulnerability to potential supply disruption, the United States (U.S.) has labeled REEs as “critical minerals”.^2^ As a result, there have been increasing interest and research to explore alternative REE resources and recovery of REEs from waste streams. For example, the U.S. Department of Energy has initiated programs to examine methods of recovering REEs from coal-related wastes.^3^ Coal fly ash (CFA) has been proposed as a promising resource for REE recovery.^4−8^ CFA is a sizeable industrial waste stream in the U.S., with massive reserves in legacy disposal sites plus ∼40 million tons of newly produced CFA every year.^9^ It has been estimated that only less than 40% of CFA is beneficially utilized in the U.S. in 2018, mainly in concrete production and flue gas desulfurization, and the remaining fraction is either disposed in surface impoundments or landfilled.^10,11^ Despite recent interests in research, resource recovery from CFA has yet to be practiced at scale.^12,13^ The annual value of REEs derived from CFA is estimated to be $4.3 billion.^6^ In addition, with stricter government regulations and increasing economic costs on CFA disposal,^14^ the management of CFA poses significant environmental and financial burdens. Thus, recovering REEs from CFA is a promising solution to the REE scarcity challenge and an opportunity to address the solid waste management problem.

Although REE-rich CFA was previously identified (e.g., total REEs at 17,026 ppm),^4^ CFA is typically a low-grade REE feedstock. The total REE concentration in CFA generally ranges from 250 to 800 ppm,^6^ well below the cutoff grade of 1000 ppm (expressed as rare earth oxide) suggested by Seredin and Dai.^4^ Previous studies generally focused on REE recovery and utilized highly corrosive solutions to leach REEs from CFA to yield a high REE leaching efficiency.^12,15−19^ For example, Taggart et al. sintered CFA with a 1:1 NaOH:ash ratio at 450 °C, followed by leaching with 2 M HNO_3_, achieving >70% REEs leaching from different types of CFA.^15^ To maximize the extraction efficiency, these leaching processes often require heating and highly concentrated mineral acids (e.g., 15 M HNO_3_ at 90 °C), which are thus chemical- and energy-intensive and might not be economically and environmentally viable.^6,20^ Lowering the sintering temperature or using milder mineral acid resulted in a significant decrease of REE leaching efficiency.^15^ Deng et al. reported an enhanced REE extractability from CFA by combining an ultrafast electrothermal pre-activation and leaching using diluted HCl, which demonstrates a markedly lower consumption of energy and chemicals.^20^ Nevertheless, further efforts are needed to effectively separate REEs from interfering metals.

The leachate from the acid leaching process usually has complex solution chemistry with low REE concentration (e.g., total REEs <30 mg/L) and high concentration of interfering elements (e.g., Na, Al, Ca, and Fe, at 1000–14,000 mg/L).^18^ Multiple techniques have been proposed for downstream REE separation from the leachate, such as solvent extraction,^12,16^ ionic liquid,^21,22^ liquid membrane,^12^ and biosorption.^23^ Solvent extraction is widely used in REE separation,^12,16^ but working with organic liquids (e.g., kerosene) might be hazardous and unsafe due to harmful, flammable vapors.^16^ Stoy et al. achieved efficient REE leaching and separation based on the unique thermomorphic behavior of ionic liquid betainium bis(trifluoromethylsulfonyl)imide with water.^21^ However, the synthesis of ionic liquids is currently not cost-effective, and the high viscosity of ionic liquids might slow down mass transport in large-scale processes.^22^ As reviewed by Opare et al., a cost-effective and environmentally friendly approach for REE separation is yet to be achieved;^24^ thus, the opportunities remain for exploring alternative separation technologies.

Regarding REE leaching and separation, organic ligands (especially low-molecular-weight organic acids) might be an environmentally friendly option.^25^ Our previous study demonstrated that the dominant REE-bearing phases in CFA include REE oxides, REE phosphates (e.g., monazite and xenotime), apatite, etc.^26,27^ Organic ligands such as citrate can chelate with REEs and facilitate REE leaching from REE-bearing minerals.^28^ Yang et al. tested REE leaching from coal fine refuse using citric acid and suggested that citric acid was not a competitive option compared to mineral acid.^29^ In contrast, Ji et al. examined REE leaching from coal coarse refuse using a set of organic acids, including citric acid, which showed high leaching efficiency comparable to HCl at the same pH.^30^ Yet, few studies have examined the efficiency of REE leaching from CFA using organic ligands.

As for REE separation, oxalate is an organic ligand that is widely used to precipitate REEs from acidic solutions due to the low solubility of REE oxalate precipitates (e.g., *K*_sp_ = 10^–29.2^ for La_2_(C_2_O_4_)_3(s)_).^31^ Oxalate is typically added at the later stage of REE purification with a relatively high concentration of REEs and low concentration of interfering ions (e.g., Ca^2+^). Zhang et al. modeled the interaction of REEs (0.1 mM) with oxalate in the presence of interference ions (e.g., Fe^3+^, Al^3+^, and Ca^2+^ at 0.1–1 mM) at pH 1–5 and showed that REE recovery via REE-oxalate precipitation was thermodynamically favorable.^32^ However, results from Zhang et al. might not be directly applicable to acidic leachate from REE leaching, which has much lower REE concentration, and experimental results might be different from thermodynamic calculations due to kinetic limitations. The efficacy of the selective recovery of REEs from the CFA leachate using oxalate requires further investigation. Herein, we demonstrate that oxalate can be directly added to the leachate containing coexisting Ca^2+^ to selectively separate REEs over interfering metal ions without prepurifying the leachate. Additionally, REEs or REE-bearing phases only account for a minor fraction of CFA and a considerable amount of CFA solid residue remains after upstream REE leaching.^18,26,33^ CFA solid residue and wastewater production during REE separation necessitate additional treatment steps from a waste management perspective. However, few studies have addressed the fate of secondary wastes after REE leaching and separation. CFA solid residues might be used to recover other valuable metals (e.g., Cu and Zn)^34^ or synthesize porous materials (e.g., zeolite),^35^ which might minimize waste production and add extra economic benefits.

The goal of this study is to develop an integrated system for concurrent REE recovery and waste reduction of CFA. Specifically, the system is consisted of three modules. In Module I, REE leaching from CFA using sodium citate was investigated. Citrate was selected due to its high chelating ability with REEs. For example, the stability constant of Y-citrate complex is 10^9.4^.^36^ In Module II, REE separation by directly adding oxalate to the leachate was examined. Behaviors of other valuable metals (e.g., Cu and Zn) were monitored. To better understand metal speciation and behavior in Modules I and II, thermodynamic calculation of aqueous speciation was conducted using PHREEQC.^37^ In Module III, to minimize liquid and solid waste production, the solid residue and wastewater from Modules I and II were combined to synthesize zeolite, a common industrial sorbent, as an additional marketable product. This treatment system is characterized with selective recovery of REEs, production of REE-rich product and zeolite, and minimal waste production. We evaluated this system on a Class F and a Class C CFA sample.

## 2 Materials and Methods

### 2.1 CFA Samples

Class F and Class C CFA samples were collected from coal-fired power plants located in the southeastern U.S. As two common representative CFAs, Class F CFA is typically produced by the combustion of bituminous and anthracite coals, with SiO_2_ + Al_2_O_3_ + Fe_2_O_3_ ≥ 70 wt %. Class C CFA is typically derived from subbituminous and lignite coals, with 50 wt % ≤ SiO_2_ + Al_2_O_3_ + Fe_2_O_3_ ≤ 70 wt %. These samples have been well characterized in previous studies and were labeled as samples F-1 and C-1, respectively.^26,33^ The concentrations of major elements were measured using X-ray fluorescence (XRF).^38^ The concentrations of trace metals, including REEs, were measured by inductively coupled plasma mass spectrometry (ICP-MS) after total digestion.^26,27^

### 2.2 REE Leaching Using Citrate

Unless otherwise specified, chemicals used in this study are all ACS grade or higher. To leach REEs, CFA samples were mixed with sodium citrate under continuous stirring (200 rpm) at room temperature. Solution was maintained at desired pH by periodic adjustment using dilute HCl and NaOH solutions. Effects of pH (2–7), citrate concentration (50–200 mM), and liquid-to-solid ratio (50–200 mL/g) on REE leaching were examined. After leaching, the solid residue and leachate (hereafter referred to as the citrate leachate) were separated by vacuum filtration (0.2 μm filters). The solid residue was rinsed with deionized water (18.2 MΩ/cm), dried at 45 °C, and weighed, whereas the citrate leachate was stored in a refrigerator for metal concentration measurement. The leaching efficiency of elements was calculated as
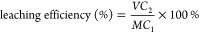
where *V* (mL) is the volume of the citrate leachate, *M* (g) is the mass of the CFA sample, and *C*_1_ and *C*_2_ (ppm) are element concentrations in the CFA sample and citrate leachate, respectively.

### 2.3 REE Separation Using Oxalate

In Module II, sodium oxalate was gradually added into the citrate leachate from Module I to separate REEs from other metals. After each addition, the suspension was allowed to react for 30 min under stirring (200 rpm) at room temperature. Solution aliquots were then collected for concentration measurement. Metals remained in the citrate leachate after oxalate addition were calculated as
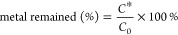
where *C*_0_ and C^*^ (ppm) are the concentration of metals in the initial citrate leachate and after oxalate addition, respectively.

The solid precipitates (hereafter referred to as the oxalate product) were harvested at the end of experiment, rinsed, and dried at 45 °C. Elemental composition of the oxalate products was measured by ICP-MS after digestion. The enrichment factor of elements in the oxalate products compared to raw CFA samples was calculated as
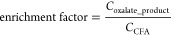
where *C*_CFA_ and *C*_oxalate_product_ (ppm) are element concentrations in the raw CFA sample and oxalate products, respectively.

### 2.4 PHREEQC Modeling

To better understand element speciation and behavior in the citrate leachate (Module I) and during oxalate precipitation (Module II), thermodynamic calculation of aqueous speciation was conducted using the program PHREEQC.^37^ The *minteq.v4.dat* database was used. Additionally, stability constants of REE–ligand complexes (e.g., Cl^–^, CO_3_^2–^, citrate, oxalate, etc.,) and solubility products of mineral phases were compiled from the literature.^36,39−46^ Citrate solution with varied concentrations of major elements (e.g., Na, Mg, Ca, and Al etc., ranging from 10 to 1000 mg/kg water) and trace elements (e.g., Cr, Co, Ni, and REEs, etc., ranging from 1 to 1000 μg/kg water) was coded in PHREEQC to mimic the citrate leachate from samples F-1 and C-1. Details of considered metal–ligand interactions and leachate chemistry are summarized in Tables S1–S4 in the Supporting Information (SI).

### 2.5 Zeolite Synthesis

To reduce the production of secondary wastes, zeolite was synthesized in hydrothermal reactors (Parr Instrument) using the solid residue from Module I after REE leaching and wastewater from Module II after REE separation. First, 0.2 g of solid residue from Module I was mixed with 10 mL of wastewater from Module II. Then, 5 M NaOH was used as an activation agent. The hydrothermal reactors were sealed and heated at 100 or 150 °C under autogenous pressure for 24 h. The synthesized solids were collected by vacuum filtration, rinsed, and dried at 45 °C for analysis.

### 2.6 Analytical Methods

#### 2.6.1 Inductively Coupled Plasma Mass Spectrometry (ICP-MS)

Element concentration in the solution during REE leaching and REE separation was measured using ICP-MS (Agilent 7500a). All solution aliquots were diluted with HNO_3_ solution and spiked with 10 ppb of indium (In) as an internal standard to monitor the instrument drift. A series of calibration standards (0–200 ppb) were prepared using standards from SPEX (CertiPrep) and Sigma-Aldrich (TraceCert). Each calibration standard was spiked with 10 ppb of In. The mass spectrometer was tuned for high sensitivity, low isobaric interference (CeO^+^/Ce^+^ <1%), and low doubly charged ions (<2%). ^53^Cr, ^59^Co, ^60^Ni, ^63^Cu, ^66^Zn, ^72^Y, ^139^La, ^140^Ce, ^141^Pr, ^146^Nd, ^147^Sm, ^153^Eu, ^157^Gd, ^159^Tb, ^163^Dy, ^165^Ho, ^166^Er, ^169^Tm, ^172^Yb, and ^175^Lu were measured. Calibration standards were measured after every 20 samples to ensure accuracy.

#### 2.6.2 X-ray Diffraction (XRD)

CFA samples, oxalate products, and zeolite products were analyzed using a Panalytical Empyrean multipurpose diffractometer with Cu Kα radiation and a PIXcel 3D-Medipi×3 1×1 detector. XRD patterns were recorded over 5–50° 2θ with a step size of 0.03° 2θ and a contact time of 15 s/step at 45 kV and 40 mA.

#### 2.6.3 Scanning Electron Microscopy and Energy-dispersive X-ray Spectroscopy (SEM-EDX)

Morphologies of CFA samples, oxalate products, and zeolite products were examined using a Hitachi SU8230 SEM. Samples were gently ground and dusted onto a carbon tape. SEM images were taken at 5 kV and 10 μA with a working distance of 5 mm. EDX spectra were obtained at 20 kV and 30 μA with a working distance of 15 mm using an Oxford X-MaxN EDX detector.

## 3 Results and Discussion

### 3.1 CFA Samples

The chemical compositions of samples F-1 and C-1 are summarized in Table 1. Sample F-1 is enriched in SiO_2_ (54.3%), Al_2_O_3_ (25.2%), and Fe_2_O_3_ (11.9%), while sample C-1 is relatively abundant in CaO (28.1%). The concentrations of other trace metals (e.g., Cr, Cu, and Zn) range from 20 to 200 ppm. Sample F-1 contains more Cr, Co, Ni, and Zn compared to sample C-1, except for Cu. The total REE content in sample F-1 is similar to that of sample C-1 (∼315 ppm). Among all REEs, Ce shows the highest concentration at ∼110 ppm. The total concentrations of light REEs (LREEs, from La to Sm) and heavy REEs (HREEs, from Eu to Lu plus Y) in both samples are around 230 and 80 ppm, respectively. Notably, the fraction of critical REEs (Nd, Eu, Tb, Dy, Y, and Er)^4^ in total REEs for sample F-1 (35.5%) is slightly higher than that of sample C-1 (32.1%).

**Table 1:** Chemical Composition of Raw CFA Samples and Oxalate Products

	sample F-1		sample C-1	
parameters	raw CFA	oxalate product	raw CFA	oxalate product
coal basin	Illinois Basin		Powder River Basin	
coal type	bituminous		subbituminous	
coal fly ash type	class F		class C	
SiO_2_ (wt %)^a^	54.3	0.0	36.6	0.0
Al_2_O_3_ (wt %)	25.2	1.6	18.2	0.8
Fe_2_O_3_ (wt %)	11.9	0.0	6.4	0.0
CaO (wt %)	1.6	93.0	28.1	95.7
other (wt %)	7.0	5.4	10.7	3.5
Cr (ppm)^b^	174.6 ± 3.0	60.8 ± 1.8	85.9 ± 9.9	2.7 ± 0.1
Co (ppm)	45.1 ± 7.8	0.8 ± 0.0	23.1 ± 0.0	5.2 ± 0.7
Ni (ppm)	116.8 ± 6.2	0.5 ± 0.0	57.0 ± 3.5	10.8 ± 0.9
Cu (ppm)	128.3 ± 5.1	79.5 ± 9.3	183.5 ± 8.0	79.6 ± 4.6
Zn (ppm)	169.9 ± 12.6	22.8 ± 0.5	109.4 ± 4.5	3.4 ± 0.0
La (ppm)	49.2 ± 0.8	208.6 ± 35.4	57.6 ± 0.43	121.0 ± 13.2
Ce (ppm)	102.2 ± 6.8	405.1 ± 38.6	111.0 ± 1.6	235.0 ± 21.1
Nd (ppm)	49.3 ± 0.2	205.9 ± 31.1	49.5 ± 0.3	104.3 ± 12.2
Eu (ppm)	2.1 ± 0.1	9.1 ± 0.8	3.0 ± 0.2	5.9 ± 0.8
Y (ppm)	52.8 ± 2.6	199.1 ± 30.8	44.1 ± 1.6	90.5 ± 13.4
LREEs (ppm)	224.0 ± 11.1	917.5 ± 121.8	241.3 ± 2.3	508.7 ± 51.5
HREEs (ppm)	91.4 ± 5.9	364.7 ± 54.2	78.5 ± 1.2	156.1 ± 25.0
critical REEs (ppm)	111.8 ± 5.9	488.6 ± 74.2	102.7 ± 1.2	228.1 ± 31.2
total REEs (ppm)	315.4 ± 9.9	1282.2 ± 176.0	319.8 ± 2.1	664.9 ± 76.6
critical REEs (%)	35.5 ± 0.0	38.1 ± 0.6	32.1 ± 0.1	34.3 ± 0.7

a Major element information measured by XRF or EDX.

b Concentrations of trace metal elements in raw CFA are from Liu et al. (2019) and Liu et al. (2020).

**Figure 1:**
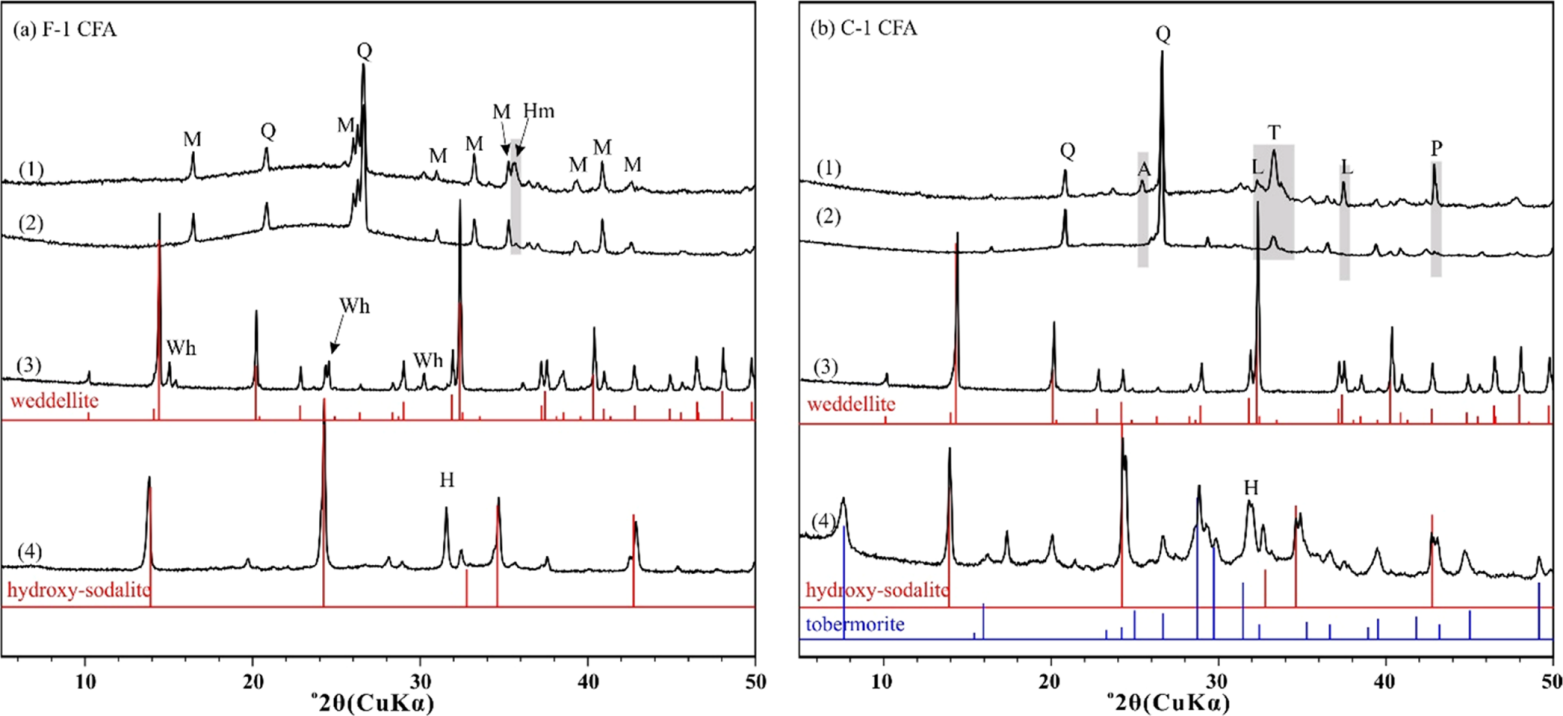
XRD patterns of the raw CFA samples and their products for (a) F-1 CFA and (b) C-1 CFA. From top to bottom: (1) raw CFA samples, (2) CFA samples after REE leaching using citrate (pH 4.0, 50 mM citrate, and liquid-to-solid ratio of 200 mL/g), (3) REE-rich oxalate products after REE separation using oxalate, and (4) zeolite products after hydrothermal synthesis at 150 °C. Vertical gray shadings indicate dissolved mineral phases after leaching. Red and blue bars are powder diffraction standards: hydroxy-sodalite ([Na_1.08_Al_2_Si_1.68_O_7.44_·1.8H_2_O], PDF 31-1271), tobermorite ([Ca_5_(OH)_2_Si_6_O_16_·4H_2_O], PDF 19-1364), and weddellite ([CaC_2_O_4_·2H_2_O], PDF 17-0541). Q (quartz, [SiO_2_]), M (mullite, [Al_6_Si_2_O_13_]), A (anhydrite, [CaSO_4_]), P (periclase, [MgO]), L (lime, [CaO]), T (tricalcium aluminate, [Ca_3_Al_2_O_6_]), Hm (hematite, [Fe_2_O_3_]), Wh (whewellite, [CaC_2_O_4_·H_2_O]), and H (halite, [NaCl]).

XRD patterns of the CFA samples are shown in Figure 1. Quartz [SiO_2_], mullite [Al_6_Si_2_O_13_], and hematite [Fe_2_O_3_] are the main mineral phases identified in sample F-1. Sample C-1 shows a complex mineralogical composition, including quartz, anhydrite [CaSO_4_], tricalcium aluminate [Ca_3_Al_2_O_6_], lime [CaO], and periclase [MgO]. Notably, a broad hump at 20–30° 2θ suggests the presence of amorphous aluminosilicate glass, which is a major component (50–80 wt %) in CFA.^47^ Under SEM, most CFA particles are spherical with a particle size of 1–100 μm (Figure S1).

### 3.2 REE Leaching Using Citrate

The leaching kinetics were first investigated using 50 mM citrate at pH 4 and a liquid-to-solid ratio of 200 mL/g. As shown in Figure S2, all metals of interest (including REEs) in samples F-1 and C-1 reached a plateau after ∼3 and ∼4 h, respectively. Based on this data, the following leaching experiments were conducted for 4 h.

**Figure 2:**
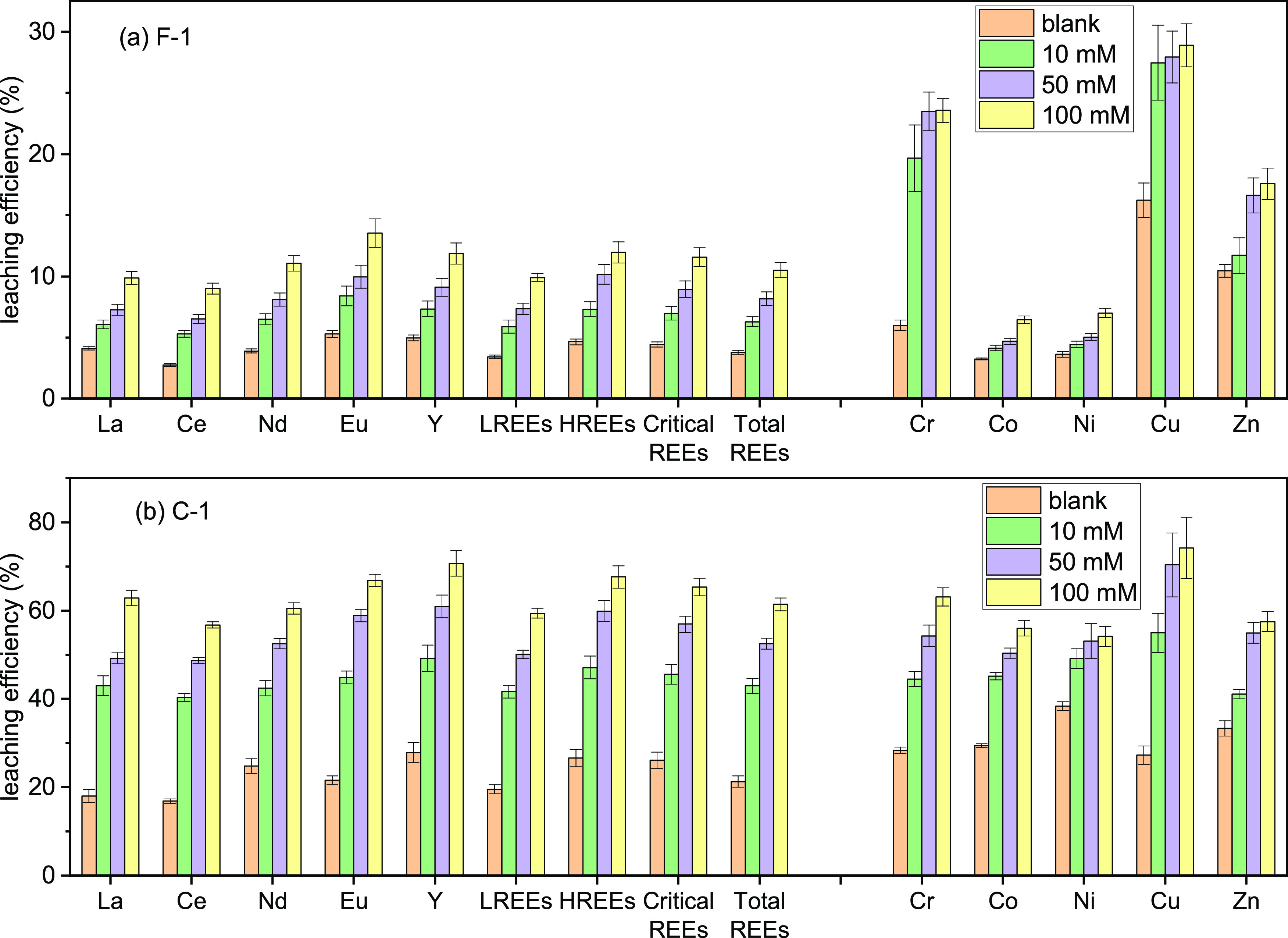
Influence of citrate concentration on metal leaching from (a) F-1 and (b) C-1 CFA samples. Leaching condition: 4 h, pH 4, liquid-to-solid ratio of 200 mL/g, and citrate concentrations at 0 (blank), 10, 50, and 100 mM; room temperature.

To examine the effect of the citrate concentration on REE leaching from CFA samples, the citrate concentration was varied (0–100 mM), while the pH and liquid-to-solid ratio were fixed at 4 and 200 mL/g, respectively. In the absence of citrate, only ∼5% of REEs were leached from sample F-1, while sample C-1 was characterized with a higher REE leaching efficiency of ∼20% (Figure 2). Previous studies also showed a higher REE leaching efficiency of Class C than Class F CFA using HNO_3_ or HCl.^6,26^ Similar behavior was also observed for other trace metals, such as Cr, Co, and Ni.^33^ However, the metal leaching efficiency of both CFA samples was low or middling at pH 4 in the absence of citrate.

In the presence of citrate, metal leaching from both CFA samples was remarkably enhanced (Figure 2). When the citrate concentration increased from 0 to 50 mM, REE leaching efficiency increased from 5 to 10% for sample F-1 and from ∼20 to ∼60% for sample C-1. Leaching efficiency of other trace metals was also improved in the presence of citrate. Thermodynamic calculation using PHREEQC shows that metal-citrate complexes are the main species of all REEs (∼100%) and other trace metals (e.g., >90% for Co, Ni, and Cu) (Figure S3).

The solid residue of the CFA samples after REE leaching was characterized by SEM and XRD. Although SEM did not observe noticeable morphological changes (Figure S1), XRD analysis shows mineral dissolution after REE leaching (Figure 1). After REE leaching using citrate, hematite was completely dissolved in sample F-1, whereas anhydrite, lime, periclase, and most of the tricalcium aluminate were dissolved in sample C-1. The formation of metal-citrate complexes and dissolution of solid phases might explain the increasing metal leaching efficiency in the presence of citrate.

Further increasing the citrate concentration from 50 to 100 mM did not lead to a notable increase in metal leaching efficiency, likely reaching a maximum leaching efficiency at pH 4 (Figure 2). The influence of pH and liquid-to-solid ratio on leaching efficiency was explored (Figures S4 and S5), and the details are discussed in Text S1 to avoid redundancy.

### 3.3 REE Separation Using Oxalate

Following REE leaching (Module I), a REE separation step (Module II) to precipitate REEs from the citrate leachate was conducted. The citrate leachate (0.2 μm filters) was collected from Module I with a reaction condition of 50 mM citrate, pH 4, and liquid-to-solid ratio of 200 mL/g. This reaction condition was selected for REE separation because metal leaching efficiency is relatively high for both CFA samples at this condition and the leachate is not extremely acidic.

**Figure 3:**
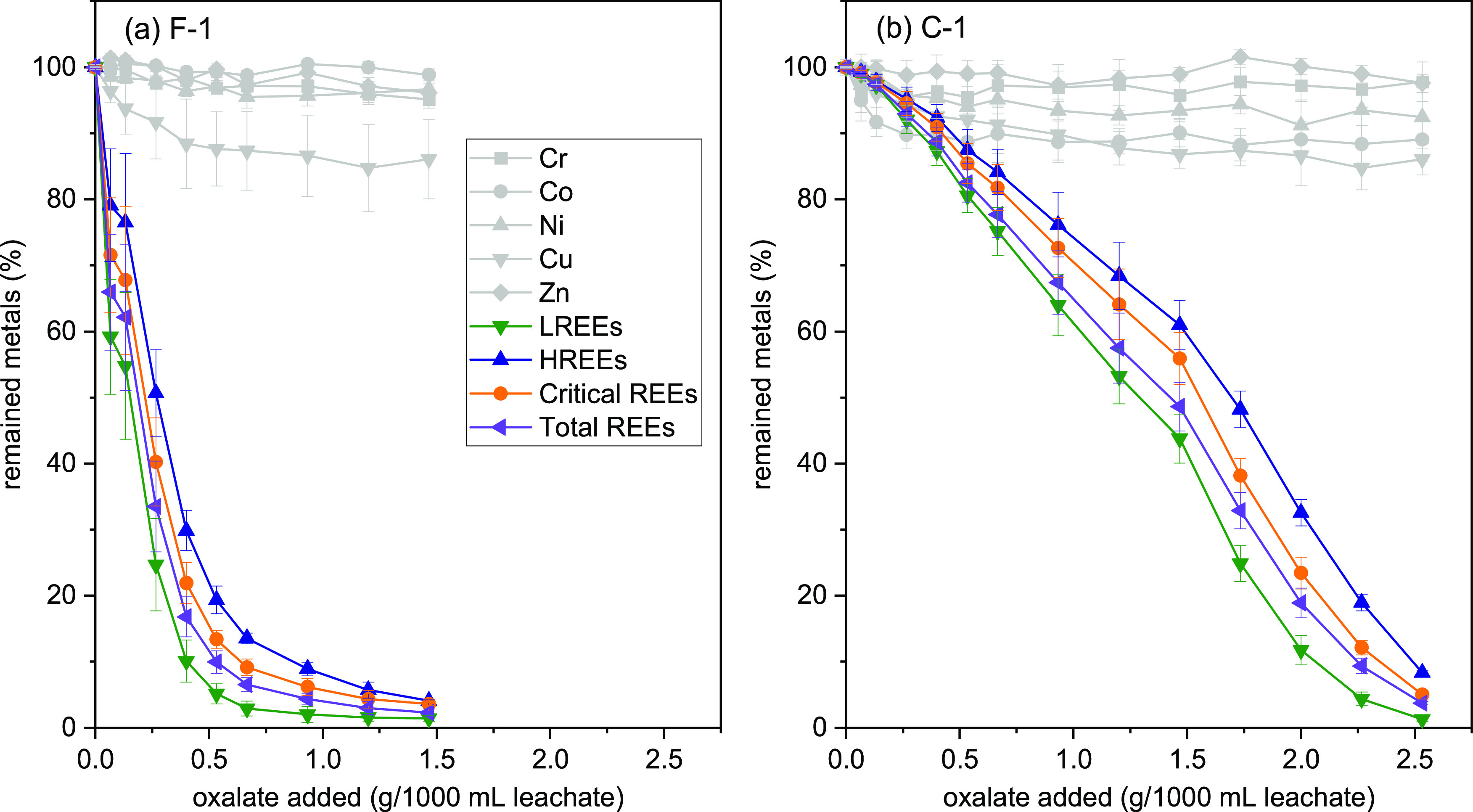
Fraction of metals remained in the citrate leachate as a function of added sodium oxalate. Leaching solutions of panels (a) and (b) are from F-1 and C-1 CFA samples, respectively. After each oxalate addition, the whole system was allowed to react for 30 min.

The fraction of metals remained in the solution as a function of added oxalate is plotted in Figure 3. A sharp decrease in the concentration of dissolved REE with the addition of oxalate and formation of precipitates was observed (hereafter referred to as the oxalate product), while other metals (Cr, Co, Ni, Cu, and Zn) remained in the solution with only up to ∼10% removal (e.g., Cu). In addition, the decrease of REE concentration for sample F-1 was significantly faster than that of sample C-1. As a result, only ∼1.5 mg oxalate (per 1000 mL leachate) is needed to precipitate >95% REEs from the citrate leachate of sample F-1, and ∼2.5 mg oxalate (per 1000 mL leachate) is required for sample C-1 (Figure 3). The remained fractions of individual REEs in the solution are presented in Figure S6. During the whole process, the solution pH slightly increases from 4.00 to 4.15 (Figure S7).

XRD analysis of the precipitated solid products (Figure 1) identified the solids to be primarily weddellite [CaC_2_O_4_·2H_2_O]. For the precipitates from sample F-1, there is also a small amount of whewellite [CaC_2_O_4_·H_2_O]. Under SEM (Figure S8), weddellite particles are easily recognized by the distinctive bipyramid shape, which corresponds to the (101) facets of the tetragonal structure; whewellite particles, on the other hand, have a platelike shape, corresponding to the (100) facets.^48−50^

PHREEQC calculation suggests that oxalate outcompetes citrate for trace metals and REEs under the experimental condition, and calcium oxalate becomes the dominant solid phase as the oxalate concentration increases. For example, in the presence of 50 mM citrate, addition of ∼3.4 g oxalate in 1000 mL of citrate leachate (i.e., 20 mM oxalate) resulted in the dominant presence of a metal-oxalate complex for Cu, Zn, and REEs (Figure S9). With the continuous addition of oxalate, calcium oxalate is the only phase that is oversaturated and predicted to precipitate (i.e., saturation index, SI > 0), which is in line with the XRD and SEM results. In contrast, the precipitation of oxalate with REEs or other metals (e.g., Mg, Sr, Cu, and Zn) is not thermodynamically favorable (i.e., SI <0). The decrease of Ca concentration in F-1 and C-1 leachate calculated by PHREEQC (Figure S10) showed a trend similar to the decreases of REEs (Figure 3). Such a consistency might suggest the incorporation or coprecipitation of REEs during the formation of calcium oxalate (mainly weddellite). Moreover, the preferential incorporation of REEs over other metals (e.g., Cr, Co, Ni, Cu, and Zn) might be explained by the fact that the cation radii of REEs are more similar to Ca^2+^ in weddellite (Table S5). In the lattice of weddellite, the Ca coordination polyhedron consists of eight oxygen atoms, six of which are from four oxalate groups and the remaining two from two water molecules.^48^ The effective cation radius of Ca is 1.12 Å with a coordination number of 8, which is similar to the cation radii of REEs ranging from 1.160 to 0.972 Å with the 8-fold coordination,^51^ while the cation radii of other metals (Cr, Co, Ni, Cu, and Zn) are much smaller (0.90 Å) or do not allow 8-fold coordination,^51^ and therefore, these metals are less likely to incorporate in weddellite (Table S5).

**Figure 4:**
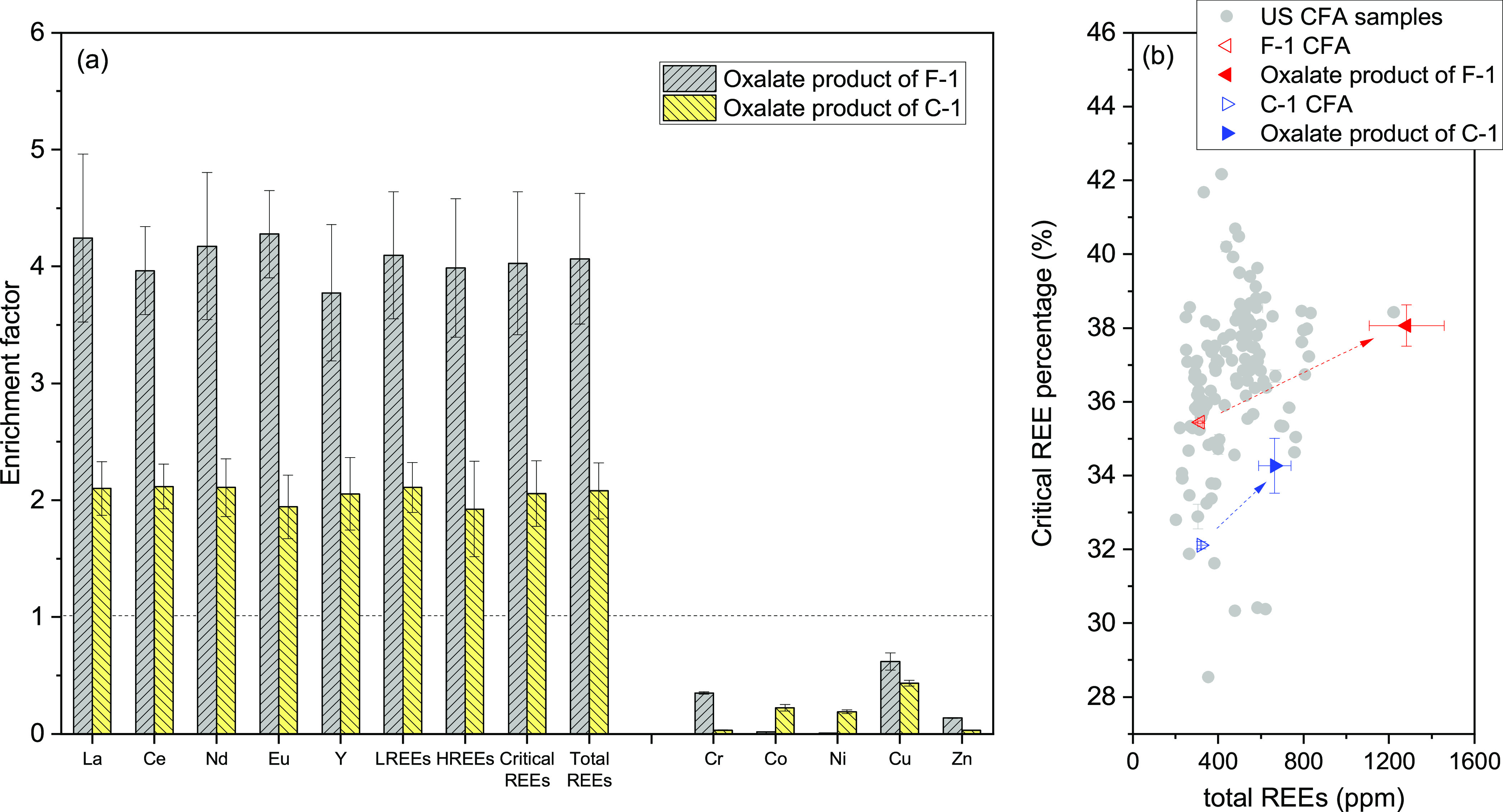
(a) Enrichment factor of metals in oxalate products compared to their corresponding concentrations in raw CFA samples F-1 and C-1. (b) Percentage of critical REEs (Nd, Eu, Tb, Dy, Y, and Er) vs total REEs of raw CFA samples and oxalate products. Gray points in panel (b) are summarized United States (U.S.) CFA samples from Taggart et al.^6^

Metal concentrations in the oxalate product are summarized in Table 1, and the enrichment factors of metals are plotted in Figures 4 and S11. The oxalate product is depleted in Cr, Co, Ni, Cu, and Zn compared to the raw CFA samples (i.e., enrichment factor <1). In marked contrast, REEs are substantially enriched in the oxalate product. The total REE concentration in the oxalate product from sample F-1 is close to 1300 ppm, which is 4 times higher than that of the raw sample F-1. For the product from sample C-1, this value is about 650 ppm, 2 times higher than the raw C-1 CFA sample. Such enrichment factors are more efficient than or comparable to physical enrichment methods (e.g., 2.14 for density separation),^52^ combined physical separation, and hydrothermal enrichment methods at 2.7 for Class F CFA,^53^ solvent extraction at 2.6, and liquid membrane at 2.4–7.5 for Class F CFA (calculated based on reported results by Smith et al.^12^). EDX spectra show the predominance of CaO (>93 wt %) in the oxalate product (Table 1). REEs are not detected in the oxalate product, probably because the concentration of individual REE is still below the EDX detection limit (∼0.1 wt %). The percentage of critical REEs in total REEs for the raw CFA samples and REE-rich oxalate product is plotted in Figure 4b and compared to U.S.-based CFA samples.^6^ Most U.S.-based CFA samples contain 32–38% critical REEs, and the total REEs range from 250 to 800 ppm (points fall in the bottom-left of Figure 4b). After metal leaching using citrate and metal precipitation using oxalate, data points of oxalate product shift toward the upper-right corner. The REE-rich oxalate product is more promising for downstream REE recovery as they display enhanced total REE concentration and percentage of critical REEs and less interfering metals.

To evaluate the environmental impacts of coextracted heavy metals from CFA, their concentrations in the citrate leachate and oxalate filtrate are summarized in Table S6. Although the concentrations of Cr are slightly higher than the EPA primary drinking water standard (100 ppb), it can be effectively removed by conventional processes such as coagulation/flocculation and ion exchange. On the other hand, the concentrations of Cu are well below its regulatory limit (1300 ppb). For Co and Ni that are not regulated by EPA, their concentrations are relatively low. Thus, we suggest that the environmental impacts of these coextracted heavy metals are low.

### 3.4 Zeolite Synthesis

Zeolite is a group of crystalline aluminosilicate minerals, which has a three-dimensional framework of Si/Al tetrahedra with lots of voids and open spaces. Additionally, the substitution of Si(IV) by Al(III) results in permanent negative changes of zeolite and consequently high cation-exchange capacity (CEC).^54^ Because of those unique properties, zeolite has a wide range of industrial applications (e.g., contaminant sequestration and molecule sieve).^55^ Hydrothermal synthesis of zeolite from CFA has been extensively studied. About 15 types of zeolite (e.g., zeolite NaP1, A, and X) can be synthesized from CFA depending on the CFA chemical composition (e.g., SiO_2_/Al_2_O_3_ ratio), temperature (e.g., 80–200 °C), alkaline solution concentration (e.g., 0.5–5 M NaOH), liquid-to-solid ratio (1–50 mL/g), and reaction time (3–48 h).^54,55^

**Figure 5:**
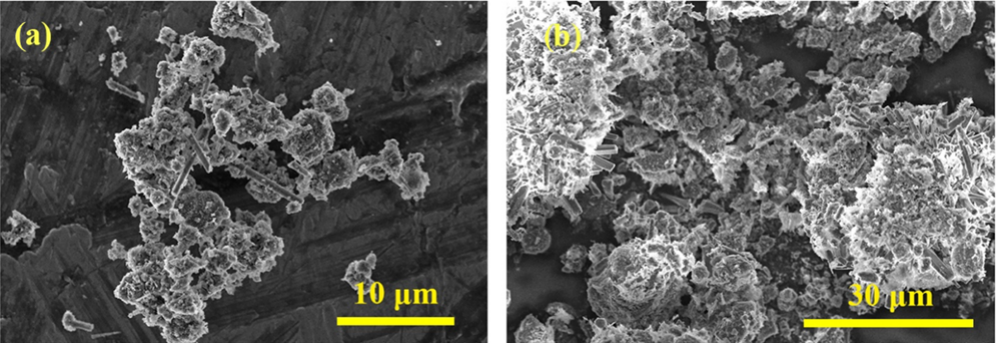
SEM images of the zeolite products after hydrothermal synthesis at 150 °C for samples (a) F-1 and (b) C-1.

To demonstrate that the CFA solid residue after leaching can be used for zeolite synthesis, the solid residue from Module I (REE leaching using citrate) was reacted with the waste leachate from Module II and 5 M NaOH at 100 or 150 °C. At 100 °C, XRD reflection peaks of quartz and mullite decreased significantly, and the broad hump at 20–30° 2θ became flattened, suggesting the disappearance of amorphous aluminosilicates (Figure S12). On the other hand, new XRD reflections of hydroxy-sodalite [Na_1.08_Al_2_Si_1.68_O_7.44_·1.8H_2_O] and halite [NaCl] appeared in both samples (Figure S11). The presence of halite in samples might be due to insufficient rinse of the products. At 150 °C, quartz and mullite completely disappeared, and more hydroxy-sodalite formed in both products (Figures 1 and S12). For sample C-1, tobermorite [Ca_5_(OH)_2_Si_6_O_16_·4H_2_O], a Ca-type zeolite, formed as well, likely due to the higher Ca content in C-1. Both hydroxy-sodalite and tobermorite are common types of zeolite that can be synthesized from CFA, especially with high concentrations of NaOH.^54^ The synthesized zeolite particles form aggregates as observed by SEM (Figure 5), which are distinctly different from the spherical morphology of CFA particles. Among zeolite particles, some rod-shaped particles (∼10 μm) are observed in both samples, which might be halite (Figure 5). The CEC of sodalite synthesized from CFA generally ranges from 250 to 350 meg/100 g,^56^ ideal for applications such as catalysis, wastewater treatment, or soil amendment.^54^

To minimize NaOH consumption and wastewater production during zeolite synthesis, the alkaline solution after one round of hydrothermal synthesis at 100 °C was tested for another round of zeolite synthesis without further addition of NaOH. It is found that the purity of zeolites was the same as the first round based on XRD analysis (Figure S13). Future experiments can be conducted to further minimize NaOH usage and optimize synthesis conditions.

### 3.5 System Analysis and Environmental Significance

The simple but effective system reported in this study combines REE leaching from CFA, preferential precipitation of REEs as a REE-rich oxalate product, and zeolite synthesis using the solid and liquid wastes from upstream steps. Compared to raw CFA samples, the oxalate product is 2–4 times more enriched in REEs (especially critical REEs) and contains less impurity elements such as Cr, Co, and Cu. Therefore, the oxalate product could be used as a more promising REE feedstock for a downstream REE purification process (e.g., electrodeposition and calcination) to yield single REE products. As for zeolite, previous studies showed that the purity of zeolite from CFA varied widely at 40–75%, depending on the contents of glass phases, nonreactive phases (e.g., hematite and lime), and resistant silicates (e.g., quartz and mullite).^54^ This study produced near-pure zeolite products at 150 °C, given that halite can be easily removed by washing. The REE leaching experiment using citrate might serve as a pretreatment process to remove nonreactive phases (such as hematite, lime, and periclase) (Figure 1) prior to hydrothermal synthesis and thus resulted in the high purity of zeolite in this study.^57^ Importantly, as all CFA solid residues are converted to zeolite, no solid waste will be produced from this system from a perspective of solid waste management. Wastewater from zeolite synthesis could be reused to minimize wastewater production (Figure S12). By completely converting CFA to REE-rich oxalate products and zeolite, this approach can bring about great economic and environmental benefits.

The proposed system could be optimized in the future to maximize economic and environmental benefits. For example, the citrate concentration might be tailored for Class F vs Class C CFA to minimize citrate consumption (Figure 2). The remaining leachate after REE separation is promising for recovering other valuable metals (e.g., Cr, Co, Ni, Cu, and Zn) (Figure 3). Cu and Zn could be preferentially precipitated by adding NaS_2_ and adjusting pH.^58^ Hydrothermal synthesis could be adjusted to sythesize high-end zeolite and to minimize NaOH consumption.^54^ Moreover, instead of purchasing citrate and oxalate directly, microbially produced citrate and oxalate might be used to reduce chemical cost. For example, *Aspergillus niger* is capable of producing citric acid or oxalic acid depending on culture medium pH, Mn availability, nitrogen limitation, etc.^59−61^

Although this system is currently tested at the bench scale, the leaching, separation, and hydrothermal synthesis steps are mature, commercially available techniques. Thus, this system is readily scalable for large-scale operation. Additionally, the proposed system might be applicable for other REE-bearing feedstock, such as weathered CFA in legacy disposal sites, coal rejects, and municipal solid waste incineration ash. Overall, the proposed approach addresses both resource recovery and solid waste management challenges with CFA. From the resource recovery aspect, this system is characterized with the production of REE-rich oxalate and zeolite. From the solid waste management aspect, the system can achieve maximum waste volume reduction and minimal production of wastewater.

## Supporting Information Available

Texts for metal leaching from CFA using citrate; tables for metal–ligand interactions and solution chemistry used in PHREEQC; and Figures for SEM-EDX images, metal leaching efficiency, metal speciation, pH evolution, and XRD patterns (PDF)
