# Supplementary material for: Green Approach for Rare Earth Element (REE) Recovery from Coal Fly Ash
Source: Environ Sci Technol. 2023 Mar 21;57(13):5414–23. doi: 10.1021/acs.est.2c09273 (PMC10077585; doi:10.1021/acs.est.2c09273)
Supplement: Supplementary file 1 — es2c09273_si_001.pdf [file es2c09273_si_001.pdf]

Supporting Information

A green approach for rare earth element (REE) recovery from coal fly ash

Pan Liu[1], Simin Zhao[1], Nan Xie[1], Lufeng Yang[2], Qian Wang[1], Yinghao Wen[1], Hailong Chen[2], Yuanzhi Tang[1]*

[1]School of Earth and Atmospheric Sciences, Georgia Institute of Technology, 311 Ferst Dr, Atlanta, GA 30332, USA

[2]Woodruff School of Mechanical Engineering, Georgia Institute of Technology, 771 Ferst Dr, Atlanta, GA 30332, USA

* Corresponding author. Email: yuanzhi.tang@eas.gatech.edu; Phone: 404-894-3814

Total 23 pages

1 text

6 tables

12 figures

Text S1. Effects of pH and liquid-to-solid ratio on metal leaching using citrate

Effect of pH. The effect of pH on metal leaching was investigated with 10 mM citrate and liquid-to-solid ratio of 200 mL/g. REE leaching efficiency increases significantly as pH decreases (Fig. S4): for sample F-1, REE leaching efficiency increases from ~5% to 10% as pH decreases from 7 to 2; while sample C-1 is characterized with a more evident increase from 20% at pH 7 to 75% at pH 2. As for other metals of interest, sample C-1 displays an evident increase from ~20% to ~70% for Cr, Co, Ni, Cu, and Zn. On the other hand, Cr, Cu, and Zn in sample F-1 show an intermediate leaching efficiency of 10–20% even at pH 7 and a further increase by ~5% as pH decreases to 2, while Co and Ni in sample F-1 display a low leaching efficiency at 5% and barely increase with decreasing pH.

Effect of liquid-to-solid ratio. To explore the influence of liquid-to-solid ratio on metal leaching efficiency, pH and citrate concentration were fixed at 4.0 and 50 mM, respectively (Fig. S5). For sample F-1, increasing liquid-to-solid ratio from 50 to 100 mL/g does not result in significant change of metal leaching efficiency, and further increasing liquid-to-solid ratio to 200 mL/g only leads to 5–10% increase in leaching efficiency. In contrast, sample C-1 shows a significant increase of 10–20% in leaching efficiency for all metals as liquid-to-solid ratio increases from 50 to 100 mL/g; but further increasing of liquid-to-solid ratio to 200 mL/g does not result in a significant change.

Table S1. Logarithm of the stability constants of REE-ligand complexes, log(K), and the solubility products of REE minerals, log(K_{sp}), considered in this study. Ln^{3+} represents trivalent REEs.

Reference	Reaction equation	Y	La	Ce	Pr	Nd	Sm	Eu	Gd	Tb	Dy	Ho	Er	Tm	Yb	Lu
Luo and Byrne, 2001[1]	$Ln^{3+} + Cl^{-} = LnCl^{2+}$	0.65	0.65	0.65	0.65	0.65	0.65	0.65	0.65	0.65	0.65	0.65	0.65	0.65	0.65	0.65
Klungness and Byrne, 2000[2]	$Ln^{3+} + OH^{-} = LnOH^{2+}$	6.20	5.19	5.66	5.68	5.82	6.16	6.24	6.17	6.36	6.41	6.44	6.48	6.61	6.76	6.73
Luo and Byrne, 2004[3]	$Ln^{3+} + HCO_3^{-} = Ln(HCO_3)^{2+}$	2.32	2.34	2.31	2.25	2.28	2.34	2.47	2.36	2.46	2.50	2.46	2.49	2.52	2.53	2.49
	$Ln^{3+} + CO_3^{2-} = LnCO_3^{+}$	7.48	6.73	7.06	7.23	7.28	7.46	7.48	7.39	7.46	7.56	7.55	7.61	7.68	7.81	7.75
	$Ln^{3+} + 2CO_3^{2-} = Ln(CO_3)_2^{-}$	12.63	11.30	11.76	12.08	12.17	12.53	12.63	12.48	12.78	12.91	13.00	13.12	13.27	13.30	13.37
Schijf and Byrne, 2004[4]	$Ln^{3+} + SO_4^{2-} = LnSO_4^{-}$	3.5	3.61	3.61	3.62	3.60	3.63	3.64	3.61	3.59	3.57	3.54	3.51	3.48	3.46	3.44
Schijf and Byrne, 2001[5]	$Ln^{3+} + C_2O_4^{2-} = Ln(C_2O_4)^{+}$	6.66	5.87	5.97	6.25	6.31	6.43	6.52	6.53	6.63	6.74	6.77	6.83	6.89	6.95	6.96
	$Ln^{3+} + 2C_2O_4^{2-} = Ln(C_2O_4)_2^{-}$	11.27	10.47	10.86	10.82	10.82	11.08	11.09	11.1	11.27	11.35	11.41	11.51	11.65	11.75	11.77
Smith et al., 2004[6]	$Ln^{3+} + C_6H_5O_7^{3-} = Ln(C_6H_5O_7)$	9.42	9.18	9.42	9.50	9.51	9.59	9.46	9.38	9.30	9.34	9.39	9.41	9.55	9.65	9.67
Spahiu and Bruno, 1995[7]	$Ln_2(CO_3)_3(s) = 2Ln^{3+} + 3CO_3^{2-}$	-32.80	-35.30	-35.10	-34.80	-34.65	-34.50	-35.00	-34.70	-34.20	-34.00	-33.80	-33.60	-33.40	-33.30	-33.00
Diakonov et al., 1998[8]	$Ln(OH)_3(s) = Ln^{3+} + 3OH^{-}$	-25.93	-22.29	-23.88	-24.38	-25.98	-25.87	-26.54	-26.89	-26.31	-25.90	-26.57	-26.57	-26.75	-26.64	-26.99
Chuang et al., 1998[9]	$Ln_2(C_2O_4)_3(s) = 2Ln^{3+} + 3C_2O_4^{2-}$	-29.29	-29.22	-30.4		-30.89	-31.35	-31.38	-31.37		-30.70		-30.04		-30.02	

Table S2. Logarithm of the stability constants of metal-ligand complexes, log(K), considered in this study. M^{n+} represents cations (e.g., H^+, Mg^{2+}, etc).

Reference	Ligand	Reaction equation	H^+	Na^+	Mg^{2+}	Al^{3+}	K^+	Ca^{2+}	Fe^{3+}	Co^{2+}	Ni^{2+}	Cu^{2+}	Zn^{2+}	Sr^{2+}
minteq.v4, Parkhurst and Appelo, 2013[10]	OH^-	$M^{n+} + H_2O = M(OH)^{n-1} + H^+$			-11.397	-4.997		-12.697	-2.187	-9.697	-9.897	-7.497	-8.997	-13.177
		$M^{n+} + 2H_2O = M(OH)_2^{n-2} + 2H^+$				-10.094			-4.594	-18.794	-18.994	-16.194	-17.794	
		$M^{n+} + 2H_2O = MO(OH)^{n-3} + 3H^+$								-32.0915				
		$M^{n+} + 3H_2O = M(OH)_3^{n-3} + 3H^+$				-16.791			-12.56	-31.491	-29.991	-26.879	-28.091	
		$M^{n+} + 4H_2O = M(OH)_4^{n-4} + 4H^+$				-22.688			-21.588	-46.288		-39.98	-40.488	
		$2M^{n+} + H_2O = M_2(OH)^{2n-1} + H^+$								-10.997				
		$2M^{n+} + 2H_2O = M_2(OH)_2^{2n-2} + 2H^+$							-2.854			-10.594		
		$3M^{n+} + 4H_2O = M_3(OH)_4^{3n-4} + 4H^+$							-6.288					
		$4M^{n+} + 4H_2O = M_4(OH)_4^{4n-4} + 4H^+$								-30.488				
	Cl^-	$M^{n+} + Cl^- = MCl^{n-1}$							1.48	0.539	0.408	0.2	0.4	
		$M^{n+} + 2Cl^- = MCl_2^{n-2}$							2.13		-1.89	-0.26	0.6	
		$M^{n+} + 3Cl^- = MCl_3^{n-3}$							1.13			-2.29	0.5	
		$M^{n+} + 4Cl^- = MCl_4^{n-4}$										-4.59	0.199	
		$M^{n+} + H_2O + Cl^- = M(OH)Cl^{n-2} + H^+$											-7.48	
	CO_3^{2-}	$M^{n+} + CO_3^{2-} = M(CO_3)^{n-2}$	10.329	1.27	2.92			3.2		4.228	4.5718	6.77	4.76	2.81
		$M^{n+} + 2CO_3^{2-} = M(CO_3)_2^{n-4}$										10.2		
	HCO_3^-	$M^{n+} + H^+ + CO_3^{2-} = MH(CO_3)^{n-1}$	16.681	10.079	11.339			11.599		12.2199	12.4199	12.129	11.829	11.539
	SO_4^{2-}	$M^{n+} + SO_4^{2-} = M(SO_4)^{n-2}$	1.99	0.73	2.26	3.89	0.85	2.36		2.3	2.3	2.36	2.34	2.3
		$M^{n+} + 2SO_4^{2-} = M(SO_4)_2^{n-4}$				4.92			5.38		0.82		3.28	
Smith et al., 2004[6]	$C_2O_4^{2-}$	$M^{n+} + C_2O_4^{2-} = M(C_2O_4)^{n-2}$	4.266	0.9	3.43	6.2	0.8	3.19	7.53	4.71	5.16	4.85	4.87	2.54
		$2M^{n+} + C_2O_4^{2-} = M_2(C_2O_4)^{2n-2}$	5.516											
		$M^{n+} + 2C_2O_4^{2-} = M(C_2O_4)_2^{n-4}$				11.37			13.81	7.15	7.58	10.23	7.69	
		$M^{n+} + 3C_2O_4^{2-} = M(C_2O_4)_3^{n-6}$				15.8			18.6					
	$HC_2O_4^-$	$M^{n+} + HC_2O_4^- = M(HC_2O_4)^{n-1}$								1.61				
		$M^{n+} + 2HC_2O_4^- = M(HC_2O_4)_2^{n-2}$								2.89				
minteq.v4, Parkhurst and Appelo, 2013[10]	$C_6H_5O_7^{3-}$	$M^{n+} + C_6H_5O_7^{3-} = M(C_6H_5O_7)^{n-3}$	6.396	1.03	4.89	9.97	1.1	4.87	13.1	6.1867	6.59	7.57	6.21	4.3367
		$M^{n+} + 2C_6H_5O_7^{3-} = M(C_6H_5O_7)_2^{n-6}$				14.8					8.77	8.9	7.4	
		$2M^{n+} + C_6H_5O_7^{3-} = M_2(C_6H_5O_7)^{2n-3}$	11.157	1.5										
		$2M^{n+} + 2C_6H_5O_7^{3-} = M_2(C_6H_5O_7)_2^{2n-6}$										16.9		
	$H(C_6H_5O_7)^{2-}$	$M^{n+} + H^+ + C_6H_5O_7^{3-} = MH(C_6H_5O_7)^{n-2}$	11.157	6.45	8.91	12.85		9.26	14.4	10.4438	10.5	10.87	10.2	8.9738
		$M^{n+} + H^+ + 2C_6H_5O_7^{3-} = MH(C_6H_5O_7)_2^{n-5}$									14.9			
	$H_2(C_6H_5O_7)^-$	$M^{n+} + 2H^+ + C_6H_5O_7^{3-} = MH_2(C_6H_5O_7)^{n-2}$	14.285		12.2			12.257		12.7859	13.3	13.23	12.84	12.4859

Table S3. Logarithm of the equilibrium constants of common cation-bearing minerals, log(K), considered in this study. Equilibrium constants of hydroxides, carbonates, chlorides, and sulfates are from the minteq.v4 database of Parkhurst and Appelo, 2013[10], while equilibrium constants of oxalates are from the CRC hand book of Haynes, 2004[11].

	**Hydroxides or carbonates**	**Chlorides or sulfates**	**Oxalates**
Na^{+}	Natron<br>$Na_2CO_3{:}10H_2O = 2Na^{+} + CO_3^{2-} + 10H_2O$, log(k) = -1.311<br>Thermonatrite<br>$Na_2CO_3{:}H_2O = 2Na^{+} + CO_3^{2-} + H_2O$, log(k) = 0.637	Halite<br>$NaCl = Na^{+} + Cl^{-}$, log(k) = 1.6025<br>Thenardite<br>$Na_2SO_4 = 2Na^{+} + SO_4^{2-}$, log(k) = 0.3217<br>Mirabilite<br>$Na_2SO_4{:}10H_2O = 2Na^{+} + SO_4^{2-} + 10H_2O$, log(k) = -1.114	
Mg^{2+}	Brucite<br>$Mg(OH)_2 + 2H^{+} = Mg^{2+} + 2H_2O$, log(k) = 16.844<br>$Mg(OH)_2$(active)<br>$Mg(OH)_2 + 2H^{+} = Mg^{2+} + 2H_2O$, log(k) = 18.794<br>Huntite<br>$CaMg_3(CO_3)_4 = 3Mg^{2+} + Ca^{2+} + 4CO_3^{2-}$, log(k) = -29.968<br>Dolomite(disordered)<br>$CaMg(CO_3)_2 = Ca^{2+} + Mg^{2+} + 2CO_3^{2-}$, log(k) = -16.54<br>Dolomite(ordered)<br>$CaMg(CO_3)_2 = Ca^{2+} + Mg^{2+} + 2CO_3^{2-}$, log(k) = -17.09<br>Nesquehonite<br>$MgCO_3{:}3H_2O = Mg^{2+} + CO_3^{2-} + 3H_2O$, log(k) = -4.67<br>Magnesite<br>$MgCO_3 = Mg^{2+} + CO_3^{2-}$, log(k) = -7.46<br>Hydromagnesite<br>$Mg_5(CO_3)_4(OH)_2{:}4H_2O + 2H^{+} = 5Mg^{2+} + 4CO_3^{2-} + 6H_2O$, log(k) = -8.766<br>Artinite<br>$MgCO_3{:}Mg(OH)_2{:}3H_2O + 2H^{+} = 2Mg^{2+} + CO_3^{2-} + 5H_2O$, log(k) = 9.6	Epsomite<br>$MgSO_4{:}7H_2O = Mg^{2+} + SO_4^{2-} + 7H_2O$, log(k) = -2.1265	$MgC_2O_4(s)$<br>$MgC_2O_4(s) = Mg^{2+} + C_2O_4^{2-}$, log(k) = -5.68
Al^{3+}	Al(OH)3(am)<br>$Al(OH)_3 + 3H^{+} = Al^{3+} + 3H_2O$, log(k) = 10.8<br>Gibbsite<br>$Al(OH)_3 + 3H^{+} = Al^{3+} + 3H_2O$, log(k) = 8.291<br>Boehmite<br>$AlOOH + 3H^{+} = Al^{3+} + 2H_2O$, log(k) = 8.578<br>Diaspore<br>$AlOOH + 3H^{+} = Al^{3+} + 2H_2O$, log(k) = 6.873	Alunite<br>$KAl_3(SO_4)_2(OH)_6 + 6H^{+} = K^{+} + 3Al^{3+} + 2SO_4^{2-} + 6H_2O$, log(k) = -1.4<br>K-Alum<br>$KAl(SO_4)_2{:}12H_2O = K+ + Al^{3+} + 2SO_4^{2-} + 12H_2O$, log(k) = -5.17<br>$Al_4(OH)_{10}SO_4$<br>$Al_4(OH)_{10}SO_4 + 10H^{+} = 4Al^{3+} + SO_4^{2-} + 10H_2O$, log(k) = 22.7<br>$AlOHSO_4$<br>$AlOHSO_4 + H^{+} = Al^{3+} + SO_4^{2-} + H_2O$, log(k) = -3.23	
Ca^{2+}	Portlandite<br>$Ca(OH)_2 + 2H^{+} = Ca^{2+} + 2H_2O$, log(k) = 22.804<br>Calcite<br>$CaCO_3 = Ca^{2+} + CO_3^{2-}$, log(k) = -8.48<br>Aragonite<br>$CaCO_3 = Ca^{2+} + CO_3^{2-}$, log(k) = -8.3	Gypsum<br>$CaSO_4{:}2H_2O = Ca^{2+} + SO_4^{2-} + 2H_2O$, log(k) = -4.61<br>Anhydrite<br>$CaSO_4 = Ca^{2+} + SO_4^{2-}$, log(k) = -4.36	$CaC_2O_4(s){:}H_2O(s)$<br>$CaC_2O_4(s){:}H_2O(s) = Ca^{2+} + C_2O_4^{2-} + H_2O$, log(k) = -8.75<br>$CaC_2O_4(s){:}3H_2O(s)$<br>$CaC_2O_4(s){:}3H_2O(s) = Ca^{2+} + C_2O_4^{2-} + 3H_2O$, log(k) = -8.32
Fe^{3+}	Goethite<br>$FeOOH + 3H^{+} = Fe^{3+} + 2H_2O$, log(k) = 0.491<br>Lepidocrocite<br>$FeOOH + 3H^{+} = Fe^{3+} + 2H_2O$, log(k) = 1.371<br>Ferrihydrite<br>$Fe(OH)_3 + 3H^{+} = Fe^{3+} + 3H_2O$, log(k) = 3.191	$Fe_2(SO_4)_3$<br>$Fe_2(SO_4)_3 = 2Fe^{3+} + 3SO_4^{2-}$, log(k) = -3.7343<br>K-Jarosite<br>$KFe_3(SO_4)_2(OH)_6 + 6H^{+} = K^{+} + 3Fe^{3+} + 2SO_4^{2-} + 6H_2O$, log(k) = -14.8<br>Na-Jarosite<br>$NaFe_3(SO_4)_2(OH)_6 + 6H^{+} = Na^{+} + 3Fe^{3+} + 2SO_4^{2-} + 6H_2O$, log(k) = -11.2	

	$Fe(OH)_{2.7}Cl_{0.3}$<br>$Fe(OH)_{2.7}Cl_{0.3} + 2.7H^+ = Fe^{3+} + 2.7H_2O + 0.3Cl^-$, log(k) = -3.04		
Co^{2+}	$Co(OH)_2$<br>$Co(OH)_2 + 2H^+ = Co^{2+} + 2H_2O$, log(k) = 13.094<br>$CoCO_3$<br>$CoCO_3 = Co^{2+} + CO_3^{2-}$, log(k) = -9.98	$CoSO_4{:}6H_2O$<br>$CoSO_4{:}6H_2O = Co^{2+} + SO_4^{2-} + 6H_2O$, log(k) = -2.4726<br>$CoSO_4$<br>$CoSO_4 = Co^{2+} + SO_4^{2-}$, log(k) = 2.8024<br>$CoCl_2{:}6H_2O$<br>$CoCl_2{:}6H_2O = Co^{2+}$ + 2Cl- + $6H_2O$, log(k) = 2.5365<br>$CoCl_2$<br>$CoCl_2 = Co^{2+} + 2Cl^-$, log(k) = 8.2672	
Ni^{2+}	$Ni(OH)_2$<br>$Ni(OH)_2$ + 2H+ = $Ni^{2+} + 2H_2O$, log(k) = 12.794<br>$NiCO_3$<br>$NiCO_3 = Ni^{2+} + CO_3^{2-}$, log(k) = -6.87	Retgersite<br>$NiSO_4{:}6H_2O = Ni^{2+} + SO_4^{2-} + 6H_2O$, log(k) = -2.04<br>$Ni_4(OH)_6SO_4$<br>$Ni_4(OH)_6SO_4 + 6H^+ = 4Ni^{2+} + SO_4^{2-} + 6H_2O$, log(k) = 32<br>Morenosite<br>$NiSO_4{:}7H_2O = Ni^{2+} + SO_4^{2-} + 7H_2O$, log(k) = -2.1449	
Cu^{2+}	$Cu(OH)_2$<br>$Cu(OH)_2 + 2H^+ = Cu^{2+} + 2H_2O$, log(k) = 8.674<br>Azurite<br>$Cu_3(OH)_2(CO_3)_2 + 2H^+ = 3Cu^{2+} + 2H_2O + 2CO_3^{2-}$, log(k) = -16.906<br>Malachite<br>$Cu_2(OH)_2CO_3 + 2H^+ = 2Cu^{2+} + 2H_2O + CO_3^{2-}$, log(k) = -5.306<br>$CuCO_3$<br>$CuCO_3 = Cu^{2+} + CO_3^{2-}$, log(k) = -11.5<br>Atacamite<br>$Cu_2(OH)_3Cl + 3H^+ = 2Cu^{2+} + 3H_2O + Cl^-$, log(k) = 7.391	Langite<br>$Cu_4(OH)_6SO_4{:}H_2O + 6H^+ = 4Cu^{2+} + 7H_2O + SO_4^{2-}$, log(k) = 17.4886<br>Brochantite<br>$Cu_4(OH)_6SO_4 + 6H^+ = 4Cu^{2+} + 6H_2O + SO_4^{2-}$, log(k) = 15.222<br>Chalcanthite<br>$CuSO_4{:}5H_2O = Cu^{2+} + SO_4^{2-}$ + 5H2O, log(k) = -2.64<br>$CuSO_4$<br>$CuSO_4 = Cu^{2+} + SO_4^{2-}$, log(k) = 2.9395<br>Antlerite<br>$Cu_3(OH)_4SO_4 + 4H^+ = 3Cu^{2+} + 4H_2O + SO_4^{2-}$, log(k) = 8.788<br>Melanothallite<br>$CuCl_2 = Cu^{2+} + 2Cl^-$, log(k) = 6.2572	$CuC_2O_4(s)$<br>$CuC_2O_4(s) = Cu^{2+} + C_2O_4^{2-}$, log(k) = -9.35
Zn^{2+}	$Zn(OH)_2$(beta)<br>$Zn(OH)_2 + 2H^+ = Zn^{2+} + 2H_2O$, log(k) = 11.754<br>$Zn(OH)_2$(am)<br>$Zn(OH)_2 + 2H^+ = Zn^{2+} + 2H_2O$, log(k) = 12.474<br>$Zn(OH)_2$(gamma)<br>$Zn(OH)_2 + 2H^+ = Zn^{2+} + 2H_2O$, log(k) = 11.734<br>$Zn(OH)_2$(epsilon)<br>$Zn(OH)_2 + 2H^+ = Zn^{2+} + 2H_2O$, log(k) = 11.534<br>$Zn(OH)_2$<br>$Zn(OH)_2 + 2H^+ = Zn^{2+} + 2H_2O$, log(k) = 12.2<br>$Zn_2(OH)_3Cl$<br>$Zn_2(OH)_3Cl + 3H^+ = 2Zn^{2+} + 3H_2O + Cl^-$, log(k) = 15.191<br>$ZnCO_3{:}H_2O$<br>$ZnCO_3{:}H_2O = Zn^{2+} + CO_3^{2-} + H_2O$, log(k) = -10.26<br>Smithsonite<br>$ZnCO_3 = Zn^{2+} + CO_3^{2-}$, log(k) = -10<br>$Zn_5(OH)_8Cl_2$<br>$Zn_5(OH)_8Cl_2 + 8H^+ = 5Zn^{2+} + 8H_2O + 2Cl^-$, log(k) = 38.5	$ZnCl_2$<br>$ZnCl_2 = Zn^{2+} + 2Cl^-$, log(k) = 7.05<br>Bianchite<br>$ZnSO_4{:}6H_2O = Zn^{2+} + SO_4^{2-} + 6H_2O$, log(k) = -1.765<br>Goslarite<br>$ZnSO_4{:}7H_2O = Zn^{2+} + SO_4^{2-} + 7H_2O$, log(k) = -2.0112<br>$ZnSO_4{:}H_2O$<br>$ZnSO_4{:}H_2O = Zn^{2+} + SO_4^{2-} + H_2O$, log(k) = -0.638<br>Zincosite<br>$ZnSO_4 = Zn^{2+} + SO_4^{2-}$, log(k) = 3.9297<br>$Zn_4(OH)_6SO_4$<br>$Zn_4(OH)_6SO_4 + 6H^+ = 4Zn^{2+} + 6H_2O + SO_4^{2-}$, log(k) = 28.4<br>$Zn_2(OH)_2SO_4$<br>$Zn_2(OH)_2SO_4 + 2H^+ = 2Zn^{2+} + 2H_2O + SO_4^{2-}$, log(k) = 7.5	$ZnC_2O_4(s)$<br>$ZnC_2O_4(s) = Zn^{2+} + C_2O_4^{2-}$, log(k) = -8.86
Sr^{2+}	Strontianite<br>SrCO3 = Sr+2 + CO_3^{2-}, log(k) = -9.27	Celestite<br>SrSO4 = Sr+2 + SO_4^{2-}, log(k) = -6.62	$SrC_2O_4(s)$<br>$SrC_2O_4(s) = Sr^{2+} + C_2O_4^{2-}$, log(k) = -6.4

Table S4. Summary of leachate chemistry in PHREEQC modeling. All elements listed here were considered in speciation calculation. For the modeling of species behavior with the addition of oxalate, only species with * were considered due to incomplete thermodynamic data (See Tables S1–S3).

Chemistry	Leachate from sample F-1	Leachate from sample C-1
pH	4.0	4.0
Temperature (°C)	25	25
CO_3^{2-} (mg/kgw)*	10	20
Cl^- (mg/kgw)*	500	500
SO_4^{2-} (mg/kgw)*	50	100
Citrate (mmol/kgw)*	50	50
Oxalate (mmol/kgw)*	0–3	0–5
Na^+ (mg/kgw)*	500	500
Mg^{2+} (mg/kgw)*	50	300
Al^{3+} (mg/kgw)	50	200
K^+ (mg/kgw)*	100	100
Ca^{2+} (mg/kgw)*	200	1000
Fe^{3+} (mg/kgw)	250	100
Co^{2+} (ug/kgw)	15	100
Ni^{2+} (ug/kgw)	40	200
Cu^{2+} (ug/kgw)*	400	1000
Zn^{2+} (ug/kgw)*	150	400
Sr^{2+} (mg/kgw)	10	100
La^{3+} (ug/kgw)*	25	200
Ce^{3+} (ug/kgw)*	50	350
Pr^{3+} (ug/kgw)	6	50
Nd^{3+} (ug/kgw)*	30	170
Sm^{3+} (ug/kgw)*	6	36
Eu^{3+} (ug/kgw)*	2	10
Gd^{3+} (ug/kgw)*	8	35
Tb^{3+} (ug/kgw)	1	5
Dy^{3+} (ug/kgw)*	6	30
Y^{3+} (ug/kgw)*	30	180
Ho^{3+} (ug/kgw)	1	5
Er^{3+} (ug/kgw)*	3	20
Tm^{3+} (ug/kgw)	1	3
Yb^{3+} (ug/kgw)*	3	15
Lu^{3+} (ug/kgw)	1	2

Table S5. Summary of effective cation radii (Å) with different coordination numbers. Data from Shannon (1976) [12].

Effective ionic radius (Å)	Coordination number				
	IV	V	VI	VII	VIII
Ca^{2+}			1.00	1.06	1.12
Cr^{3+}			0.615		
Co^{2+}	0.58	0.67	0.745		0.90
Ni^{2+}	0.55	0.63	0.690		
Cu^{2+}	0.57	0.65	0.73		
Zn^{2+}	0.60	0.68	0.740		0.90
La^{3+}			1.032	1.10	1.160
Ce^{3+}			1.01	1.07	1.143
Pr^{3+}			0.99		1.126
Nd^{3+}			0.983		1.109
Sm^{3+}			0.958	1.02	1.079
Eu^{3+}			0.947	1.01	1.066
Gd^{3+}			0.938	1.00	1.053
Tb^{3+}			0.923	0.98	1.040
Dy^{3+}			0.912	0.97	1.027
Y^{3+}			0.900	0.96	1.019
Ho^{3+}			0.901		1.015
Er^{3+}			0.890	0.945	1.004
Tm^{3+}			0.880		0.994
Yb^{3+}			0.868	0.925	0.985
Lu^{3+}			0.861		0.972

Table S6. Heavy metal concentrations in raw CFA samples, citrate leachate, and oxalate filtrate. Leachate condition: 50 mM citrate, pH 4. Oxalate precipitation condition: 0–1.5 g/L oxalate for class-F CFA, 0–2.5 g/L oxalate for class-C CFA.

**Metal concentration (ppb)**	**Cr**	**Co**	**Ni**	**Cu**
**Class-F CFA**				
Raw sample	174574.6	45080.2	116762.8	128301.9
Citrate leachate	171.6	9.3	25.9	176.3
Oxalate filtrate	163.2–171.2	9.2–9.3	24.7–25.6	149.4–176.3
**Class-C CFA**				
Raw sample	85865.3	23057.1	56971.5	183545.5
Citrate leachate	191.1	52.1	70.0	504.4
Oxalate filtrate	182.4–186.9	45.9–49.4	67.6–68.5	427.4–491.8

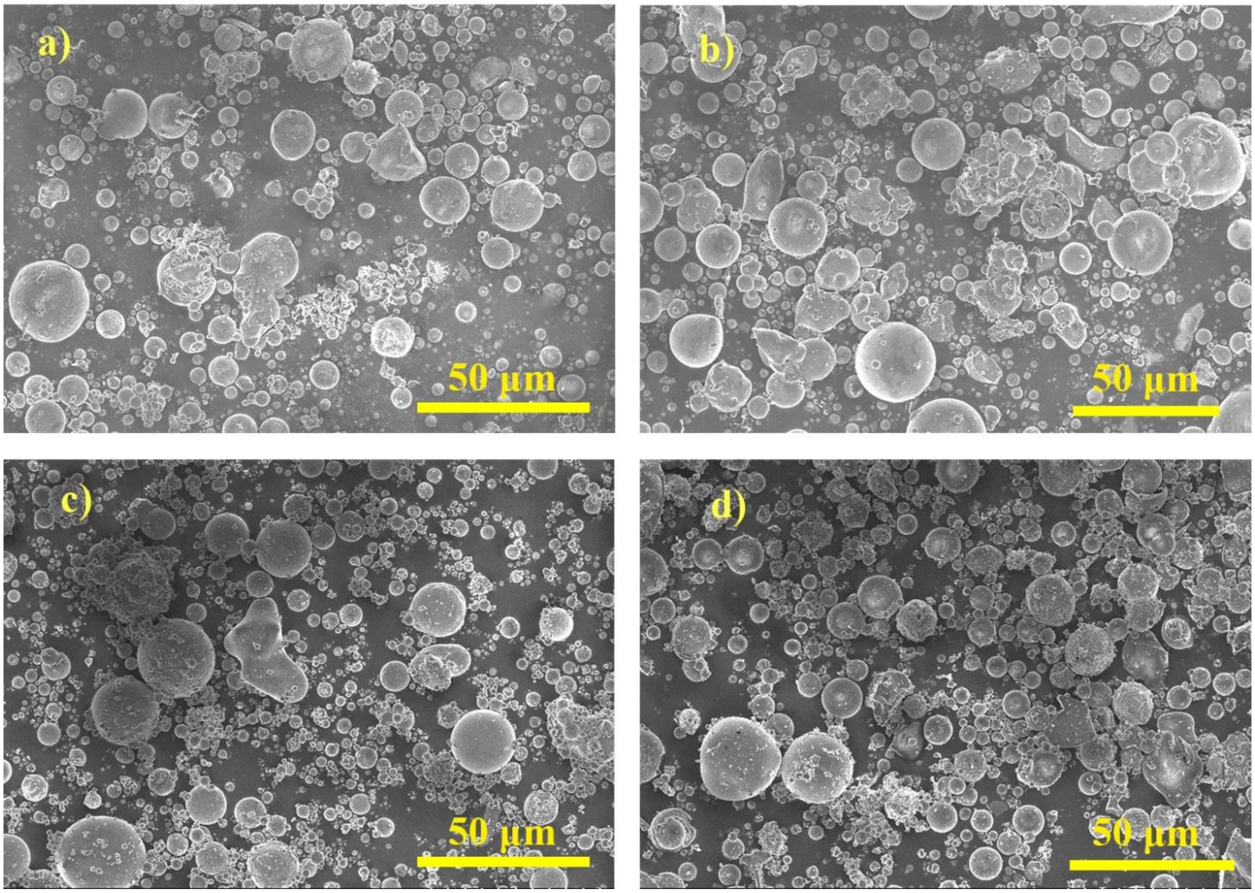

Figure S1. SEM images of CFA samples before and after REE leaching using citrate. (a) and (c) are raw F-1 and C-1 samples, respectively, while (b) and (d) are F-1 and C-1 samples after REE leaching, respectively.

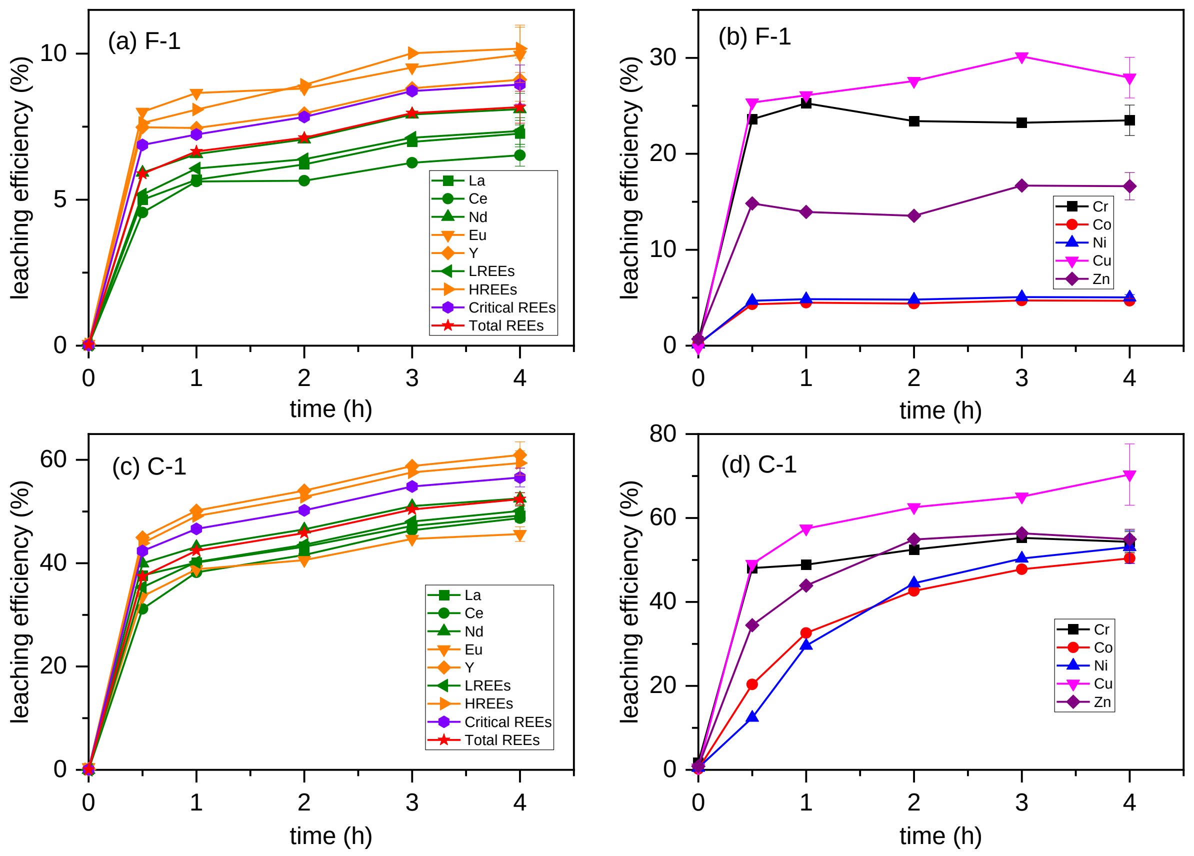

Figure S2. Kinetics of metal leaching from F-1 and C-1 CFA samples using citrate. Leaching condition: pH 4.0, 50 mM citrate, and liquid-to-solid ratio 200 mL/g.

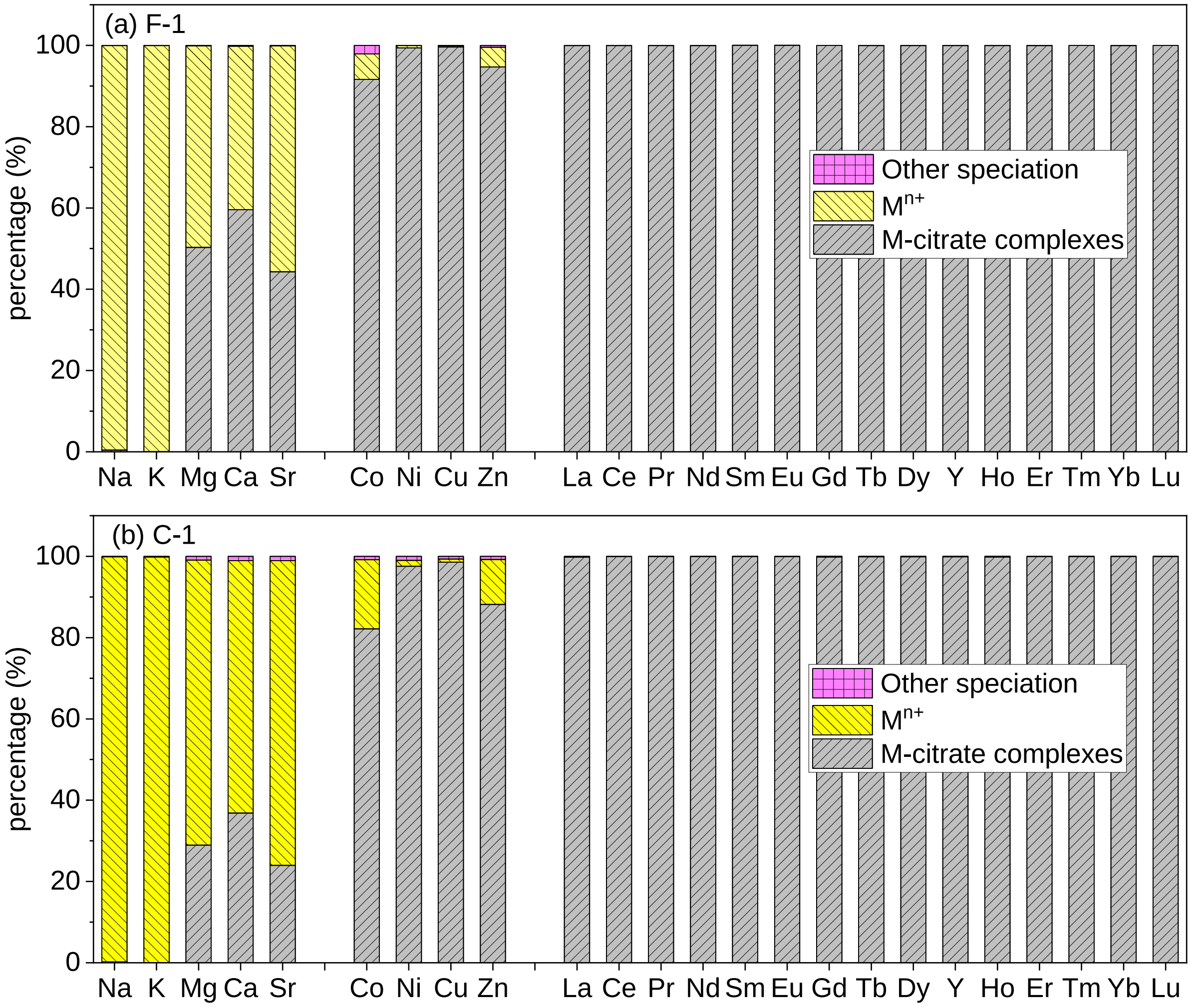

Figure S3. Metal speciation in citrate leachate modeled using PHREEQC for (a) F-1 and (b) C-1 CFA samples. *M-citrate complexes* represents metal-citrate complexes; M^{n+} denotes free metal cation; and *other speciation* includes metal-chloride, metal-sulphate complexes, etc. Refer to Tables S1–S4 for details of ligand-metal interactions and solution chemistry.

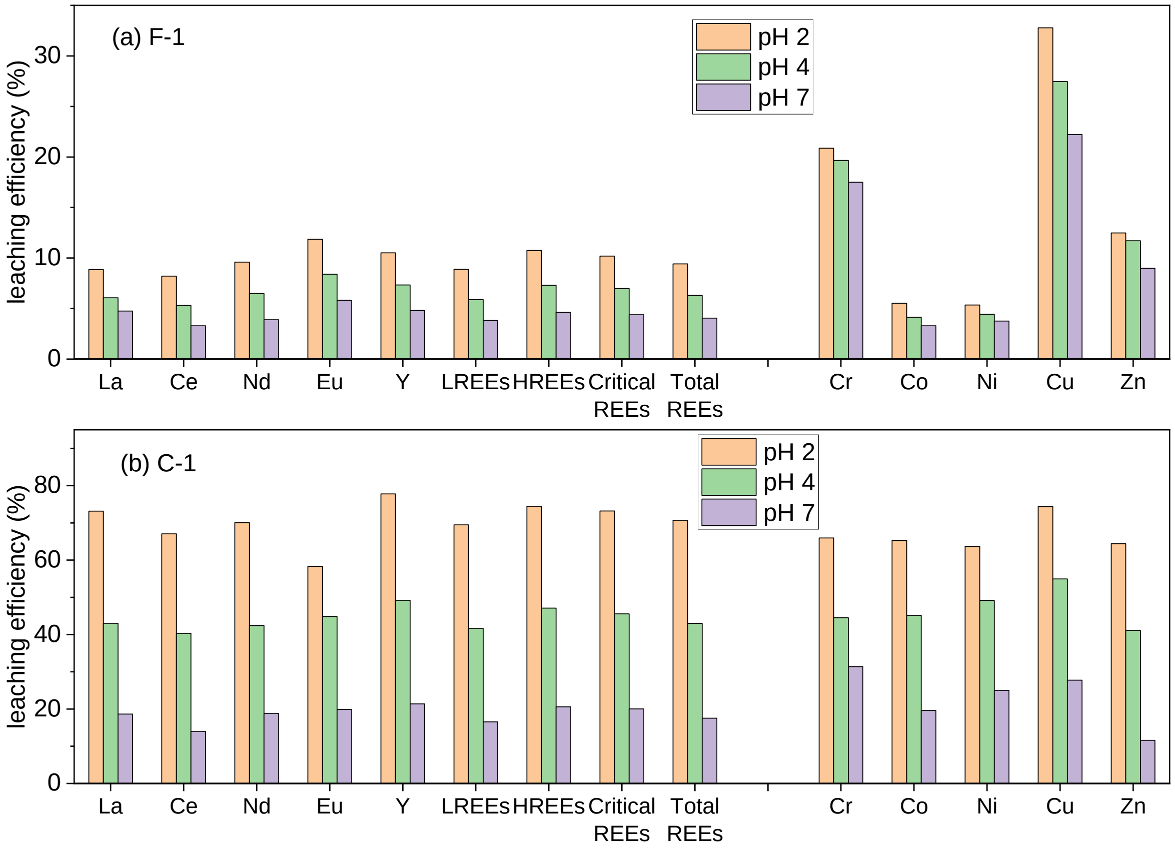

Figure S4. Influence of pH (2, 4, and 7) on metal leaching from (a) F-1 and (b) C-1 CFA samples. Leaching condition: 10 mM citrate and liquid-to-solid ratio of 200 mL/g.

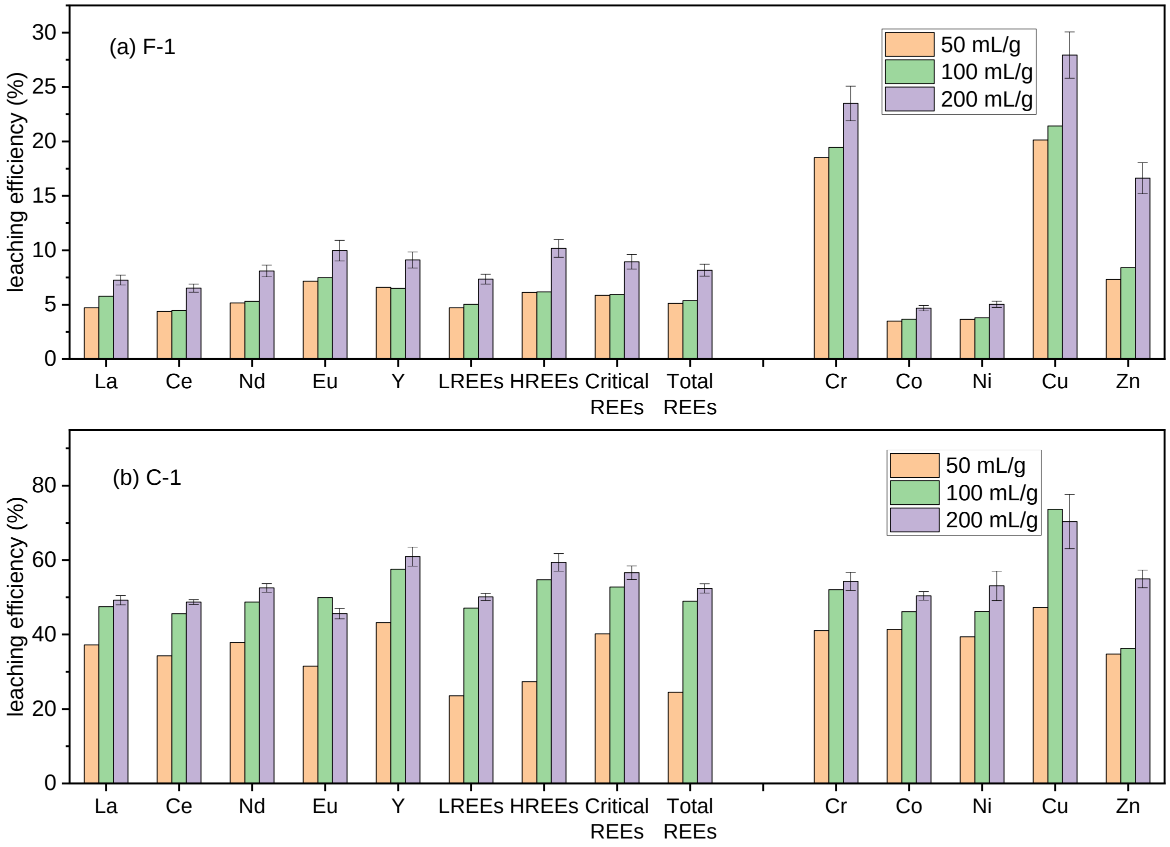

Figure S5. Influence of liquid-to-solid ratios (50, 100, and 200 mL/g) on metal leaching from samples (a) F-1 and (b) C-1. Leaching condition: pH 4 and 50 mM sodium citrate.

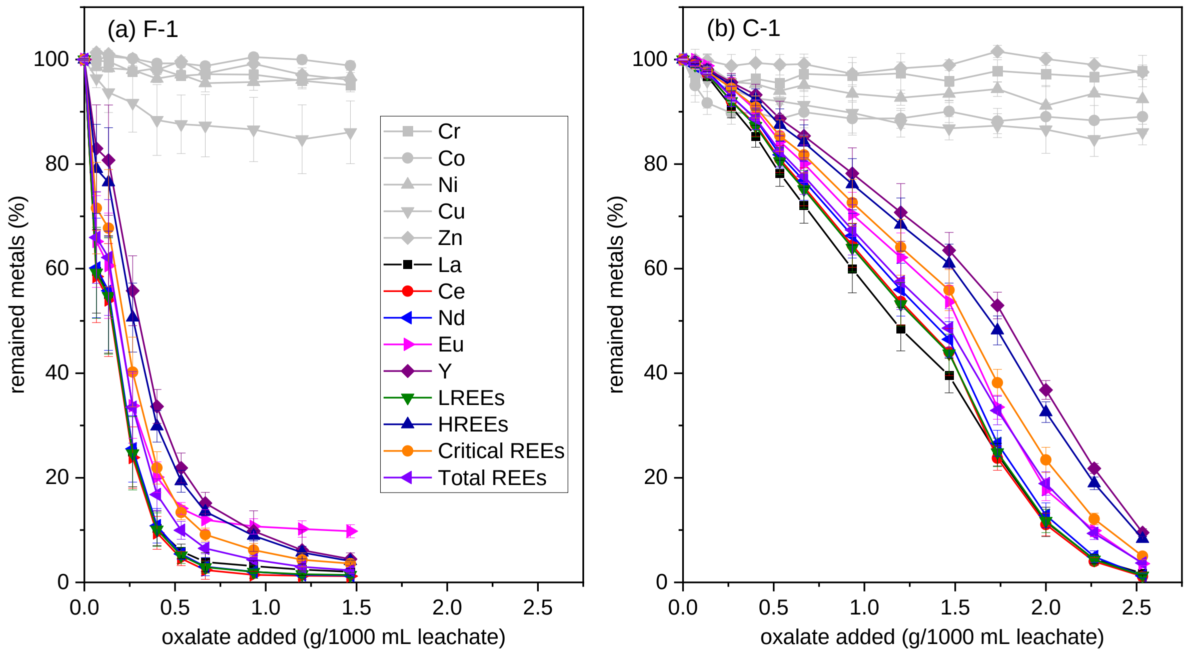

Figure S6. Fraction of metals (including individual REEs) remained in citrate leachate as a function of added sodium oxalate (duplicate). Leaching solutions of (a) and (b) are from F-1 and C-1 CFA samples, respectively. After each oxalate addition, the whole system was allowed to react for 30 min.

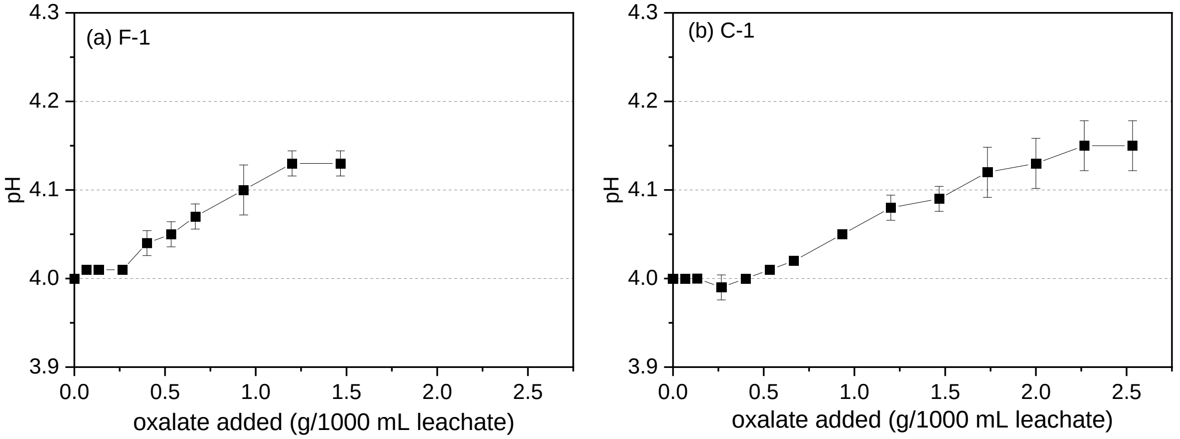

Figure S7. pH of citrate leachate as a function of added sodium oxalate. Leaching solutions of (a) and (b) are from F-1 and C-1 CFA samples, respectively. After each oxalate addition, the whole system was allowed to react for 30 min.

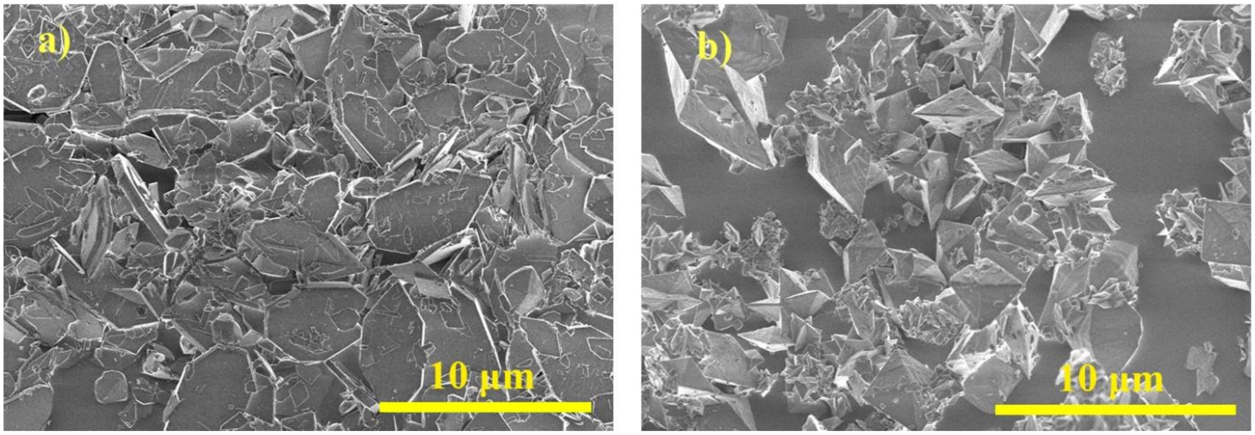

Figure S8. SEM images of the oxalate products after oxalate addition to the citrate leachate from samples (a) F-1 and (b) C-1.

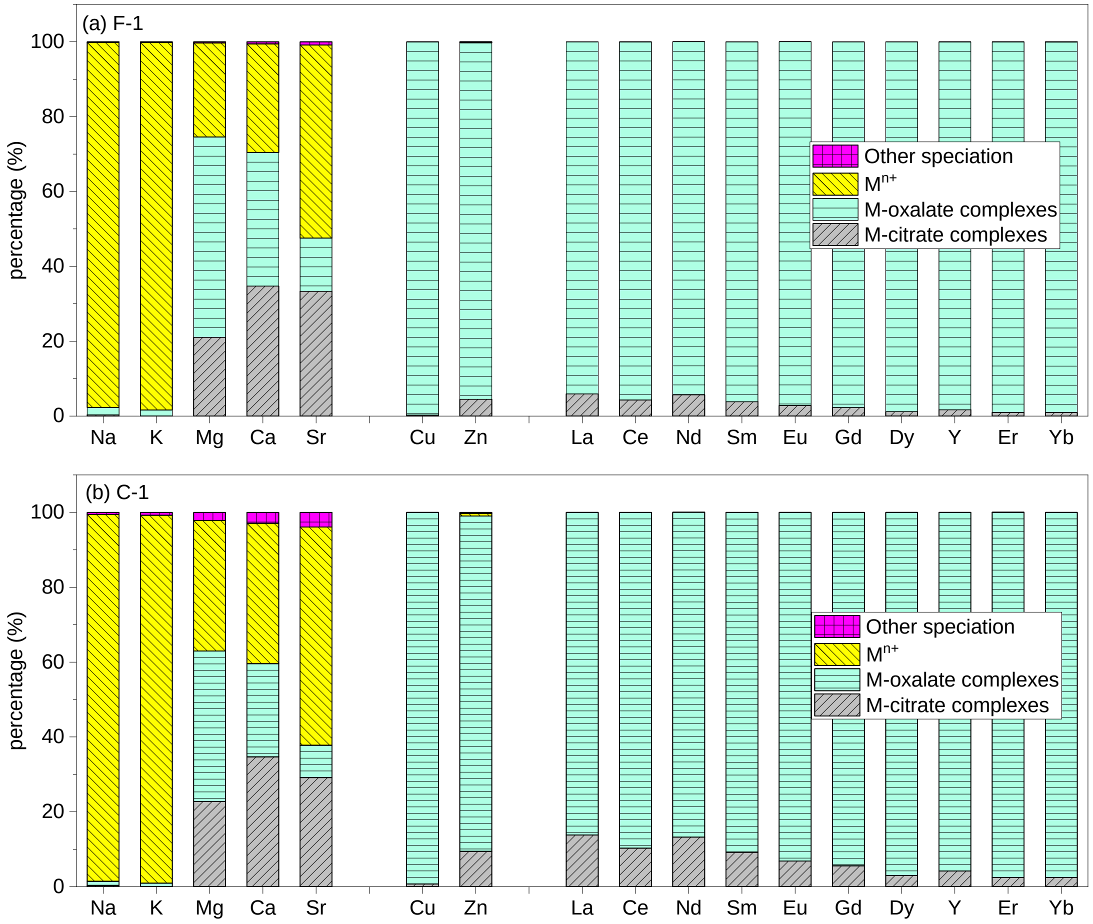

Figure S9. Metal speciation in the presence of citrate (50 mM) and oxalate (20 mM) modeled by PHREEQC for (a) F-1 and (b) C-1 CFA samples. *M-citrate complexes* represents metal-citrate complexes; *M-oxalate complexes* denotes metal-oxalate complexes; M^{n+} represents free metal cation; *other speciation* includes metal-chloride, metal-sulphate complexes, etc. Refer to Tables S1–S4 for details of ligand-metal interactions and solution chemistry.

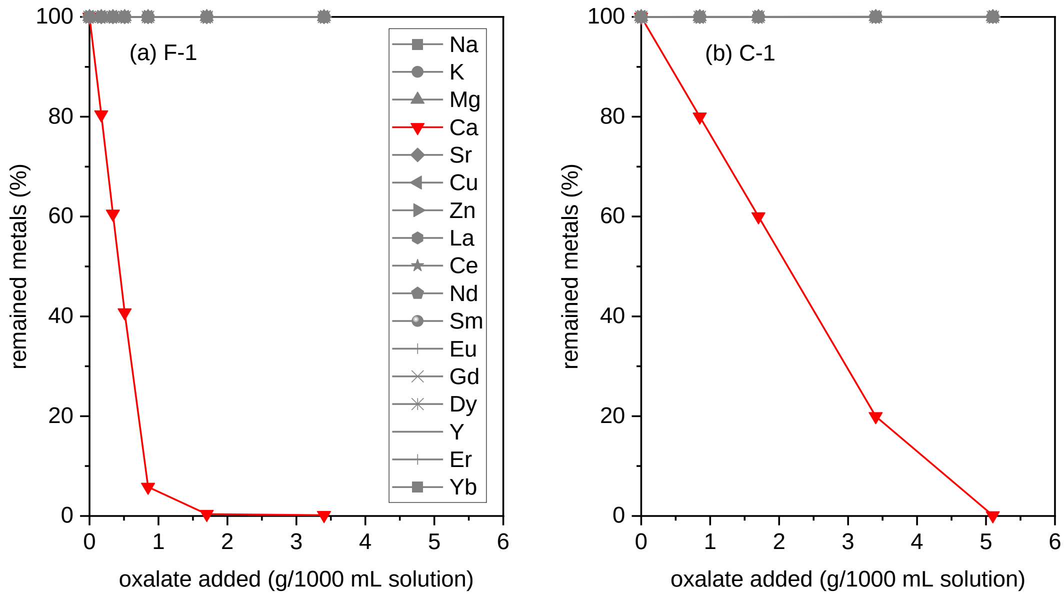

Figure S10. Evolution of metals remained in leachate as a function of added sodium oxalate modeled by PHREEQC. Leachate from samples (a) F-1 and (b) C-1. In both (a) and (b), only Ca^{2+} is predicted to precipitate from solution. Refer to Tables S1–S4 for details of ligand-metal interactions and solution chemistry.

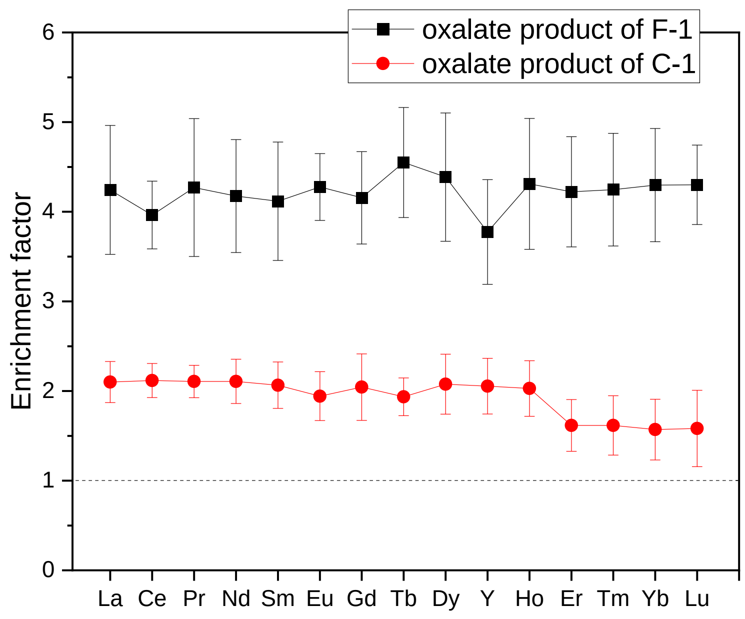

Figure S11. Enrichment factor of individual REE of the oxalate products as compared to raw CFA samples (duplicate).

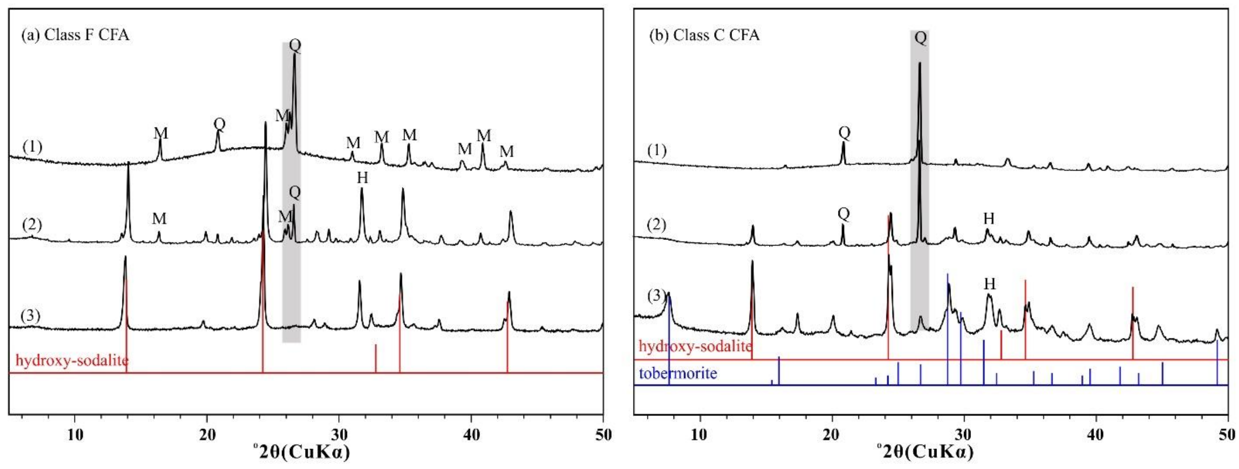

Figure S12. XRD patterns of CFA solid residue and zeolite product synthesized at different temperature. From top to bottom: (1) CFA solid residue after REE leaching using citrate, (2) zeolite product after hydrothermal synthesis at 100 °C, and (3) zeolite product after hydrothermal synthesis at 150 °C. Vertical gray shadings show the disappearance of quartz and mullite. Red and blue bars are powder diffraction standards: hydroxy-sodalite ([$Na_{1.08}Al_2Si_{1.68}O_{7.44}\cdot 1.8H_2O$], PDF 31-1271), tobermorite ([$Ca_5(OH)_2Si_6O_{16}\cdot 4H_2O$], PDF 19-1364), Q (quartz, [SiO_2]), M (mullite, [$Al_6Si_2O_{13}$]), and H (halite, [NaCl]).

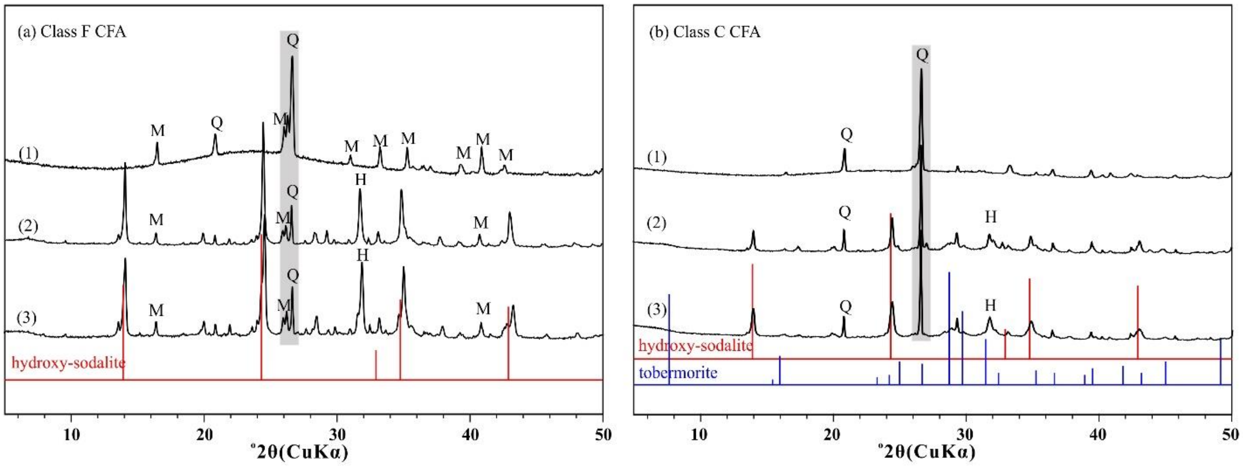

Figure S13. XRD patterns of CFA residues and zeolite products synthesized at 100 °C. From top to bottom: (1) CFA residues after metal leaching using citrate, (2) zeolite product after hydrothermal synthesis at 100 °C, and (3) zeolite product synthesized at 100 °C by reusing alkaline solution from (2). Vertical gray shadings show disappearance of quartz and mullite. Red and blue bars are powder diffraction standards: hydroxy-sodalite ([$Na_{1.08}Al_2Si_{1.68}O_{7.44}\cdot 1.8H_2O$], PDF 31-1271), and tobermorite ([$Ca_5(OH)_2Si_6O_{16}\cdot 4H_2O$], PDF 19-1364). Q (quartz, [SiO_2]), M (mullite, [$Al_6Si_2O_{13}$]), and H (halite, [NaCl]).

Reference

(1) Luo, Y.-R.; Byrne, R. H. Yttrium and rare earth element complexation by chloride ions at 25 C. *Journal of Solution Chemistry* **2001**, *30* (9), 837-845.

(2) Klungness, G. D.; Byrne, R. H. Comparative hydrolysis behavior of the rare earths and yttrium: the influence of temperature and ionic strength. *Polyhedron* **2000**, *19* (1), 99-107.

(3) Luo, Y.-R.; Byrne, R. H. Carbonate complexation of yttrium and the rare earth elements in natural waters. *Geochimica et Cosmochimica Acta* **2004**, *68* (4), 691-699.

(4) Schijf, J.; Byrne, R. H. Determination of SO4β1 for yttrium and the rare earth elements at I= 0.66 m and t= 25 C—implications for YREE solution speciation in sulfate-rich waters. *Geochimica et Cosmochimica Acta* **2004**, *68* (13), 2825-2837.

(5) Schijf, J.; Byrne, R. Stability constants for mono-and dioxalato-complexes of Y and the REE, potentially important species in groundwaters and surface freshwaters. *Geochimica et Cosmochimica Acta* **2001**, *65* (7), 1037-1046.

(6) Smith, R.; Martell, A.; Motekaitis, R. NIST standard reference database 46. *NIST Critically Selected Stability Constants of Metal Complexes Database Ver* **2004**, *2*.

(7) Spahiu, K.; Bruno, J. *A selected thermodynamic database for REE to be used in HLNW performance assessment exercises*; Swedish Nuclear Fuel and Waste Management Co., 1995.

(8) Diakonov, I.; Ragnarsdottir, K.; Tagirov, B. Standard thermodynamic properties and heat capacity equations of rare earth hydroxides:: II. Ce (III)-, Pr-, Sm-, Eu (III)-, Gd-, Tb-, Dy-, Ho-, Er-, Tm-, Yb-, and Y-hydroxides. Comparison of thermochemical and solubility data. *Chemical geology* **1998**, *151* (1-4), 327-347.

(9) Chung, D.-Y.; Kim, E.-H.; Lee, E.-H.; Yoo, J.-H. Solubility of rare earth oxalate in oxalic and nitric acid media. *Journal of Industrial and Engineering Chemistry* **1998**, *4* (4), 277-284.

(10) Parkhurst, D. L.; Appelo, C. *Description of input and examples for PHREEQC version 3: a computer program for speciation, batch-reaction, one-dimensional transport, and inverse geochemical calculations*; US Geological Survey, 2013.

(11) Haynes, W. M. *CRC handbook of chemistry and physics*; CRC press, 2014.

(12) Shannon, R. D. Revised effective ionic radii and systematic studies of interatomic distances in halides and chalcogenides. *Acta crystallographica section A: crystal physics, diffraction, theoretical and general crystallography* **1976**, *32* (5), 751-767.
